# Mortality among populations affected by armed conflict in northeast Nigeria, 2016 to 2019

**DOI:** 10.1073/pnas.2217601120

**Published:** 2023-07-19

**Authors:** Francesco Checchi, Christopher I. Jarvis, Kevin van Zandvoort, Abdihamid Warsame

**Affiliations:** ^a^Department of Infectious Disease Epidemiology, Faculty of Epidemiology and Population Health, London School of Hygiene and Tropical Medicine, London WC1E 7HT, United Kingdom

**Keywords:** mortality, Nigeria, war, humanitarian, statistical estimation

## Abstract

Armed conflict, displacement and food insecurity have affected Adamawa, Borno, and Yobe states of northeast Nigeria (population ≈ 12 million) since 2009. Insecurity escalated in 2013 to 2015, but the humanitarian response was delayed and the crisis’ health impact was unquantified due to incomplete death registration and limited ground access. We estimated mortality attributable to this crisis using a small-area estimation approach that circumvented these challenges. We fitted a mixed effects model to household mortality data collected as part of 70 ground surveys implemented by humanitarian actors. Model predictors, drawn from existing data, included livelihood typology, staple cereal price, vaccination geocoverage, and humanitarian actor presence. To project accurate death tolls, we reconstructed population denominators based on forced displacement. We used the model and population estimates to project mortality under observed conditions and varying assumed counterfactual conditions, had there been no crisis, with the difference providing excess mortality. Death rates were highly elevated across most ground surveys, with net negative household migration. Between April 2016 and December 2019, we projected 490,000 excess deaths (230,000 children under 5 y) in the most likely counterfactual scenario, with a range from 90,000 (best-case) to 550,000 (worst-case). Death rates were two to three times higher than counterfactual levels, double the projected national rate, and highest in 2016 to 2017. Despite limited scope (we could not study the situation before 2016 or in neighboring affected countries), our findings suggest a staggering health impact of this crisis. Further studies to document mortality in this and other crises are needed to guide decision-making and memorialize their human toll.

## Significance

The death toll of most wars and resulting crises, especially those in low-income countries, is poorly documented due to limited research and representative ground data. Consequently, humanitarian responders lack evidence upon which to argue for appropriate resources, and suffering and deaths remain uncounted, obfuscating historical narratives. We collected various existing datasets from the protracted crisis in northeast Nigeria, triggered by conflict between the Boko Haram group and authorities. Through statistical techniques, we estimated that some 490,900 people died as a result of this crisis during 2016 to 2019, with death rates in 2016 to 2017 more than twice Nigeria’s national average. Though our estimates feature large error margins, they illuminate the severity of this underfunded crisis and help memorialize its toll.

## The Crisis in Northeast Nigeria

A large region of north-east Nigeria and, increasingly, neighboring countries including Nigeria, Chad, and Cameroon have been affected by protracted crisis conditions linked to an armed conflict between authorities, local defense units, and the Boko Haram group. Poverty, inequality and environmental shocks (including the loss of livelihoods around Lake Chad) are considered root causes of the conflict (1–3). Within Nigeria, Adamawa, Borno, and Yobe states have been disproportionately affected. The conflict began in 2009, but insecurity escalated in 2013 and 2014, leaving 1.7 M people internally displaced by mid-2015, a 10-fold increase from 2013 (4). In terms of people reported killed, four alternative sources of violence data (*Materials and Methods*) agree that 2015 and 2016 were the peak years, while 2014 to 2015 saw most internally displaced person (IDP) movements (*SI Appendix*, Fig. S1).

The armed conflict has been marked by widely reported attacks against civilians, including systematic sexual violence, arbitrary detention, and damage to some two thirds of health facilities in the region (5). International humanitarian assistance remained minimal until 2016 (6). The response has been heavily securitized, with aid actors largely prevented from operating outside of military-controlled enclaves hosting large concentrations of displaced or forcibly relocated people (7). The situation has been chronically alarming in inaccessible areas, where some 1.2 M civilians were estimated to remain as of end of 2019, out of some 7.9 M in need across Adamawa, Borno, and Yobe (4).

During 2016 to 2018, loss of livelihoods and access to functional markets due to the crisis combined with countrywide inflation-related food price increases to greatly exacerbate preexisting food insecurity (8). As of mid-2016, about 34% of the three states’ population was projected to be in phases 3 to 5 of the Integrated Phase Classification of food and nutrition insecurity, with suspected pockets of famine (9, 10). Measles and cholera epidemics, common manifestations of forced displacement and acute malnutrition, also occurred (11).

## Scope of This Study

Armed conflicts, especially when protracted, are characterized by increased population mortality, both directly (violence) and indirectly (increased risk of disease, reduced access to healthcare) attributable to the crisis (12). Information on mortality can inform the ongoing humanitarian response by providing a measure of the gap in avertable deaths remaining to be filled, provide evidence for resource mobilization, and support conflict resolution or at least memorialize its human impact (13). Death registration in Nigeria is limited, with about 10% completeness as of 2017 (14), and the last population census took place in 2006. There was, therefore, no ready source of comprehensive mortality information with which to carry out estimation. Moreover, a considerable proportion of the affected area was inaccessible to humanitarian actors, constraining the potential representativity of a sample survey.

To fill this information gap over as long a retrospective period as possible, we sought to quantify mortality attributable to crisis conditions in Borno, Adamawa, and Yobe states using a small-area estimation analysis of previously collected or remote sensing data, obviating the need for complex or unfeasible primary data collection in such an insecure context. The analysis period was from 2016 to 2019 and estimates were stratified by month and Local Government Area (LGA or administrative level 3, equivalent to districts). While we set out to also cover the period since 2013, very limited data availability before 2016 (*Materials and Methods*), reflecting the delayed humanitarian response, constrained our analysis period. The analysis was finalized with 2 y’ delay due to the COVID-19 pandemic.

## Results

### Crude Mortality Patterns.

Throughout the paper, we express the crude death rate (CDR) and under 5 y death rate (U5DR) as deaths per 10,000 persons (CDR) or children under 5 y old (U5DR) per day (abbreviated as person-days and child-days, respectively): these units are particularly familiar to humanitarian actors (for deaths per 1,000 per year, a unit more commonly used in demography, multiply rates by 36.5). Moreover, the U5DR is distinct from and cannot be readily converted into deaths per 1,000 live births, the metric of under 5 y mortality more commonly estimated in demography: this is because the mortality surveys we relied on measure the incidence of deaths per person-time and do not collect previous birth histories, which are commonly used to estimate survival over their first 5 y of life.

**Fig. 1.**
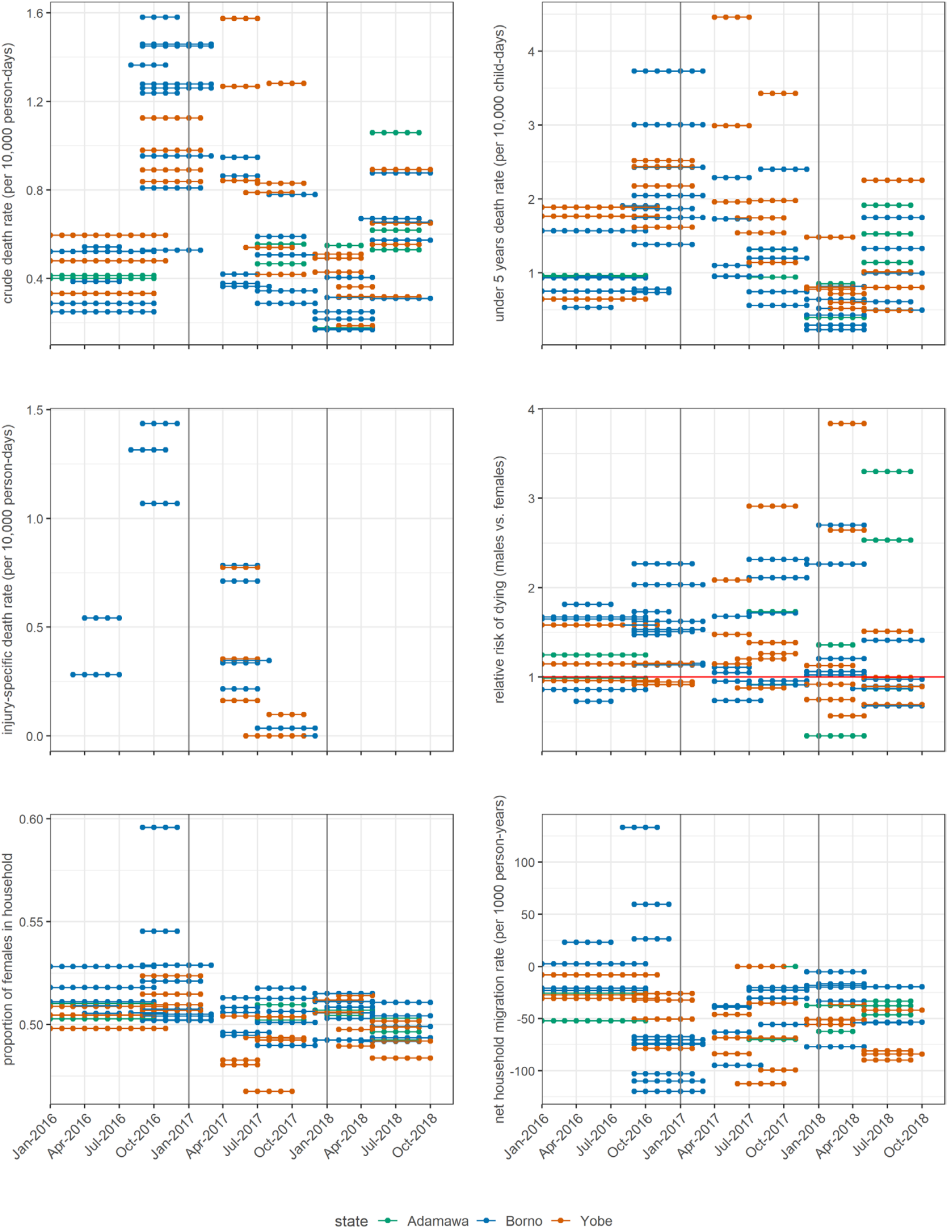
Trends in key survey-estimated demographic indicators. Each dotted segment represents the retrospective “recall” period of a single survey, namely the timespan over which household respondents were asked to report deaths and other demographic events.

Reanalysis of 70 household surveys previously conducted throughout the crisis-affected region (Table 1) showed that the CDR was generally elevated (median 0.55 per 10,000 person-days), particularly in 2017, compared to the projected median for Nigeria during 2015 to 2020 (0.34 per 10,000 person-days) (15). Both CDR and the U5DR reached peaks consistent with catastrophic emergencies (16). Injury appeared to be a leading cause of death (Table 1). While in Nigeria as a whole the ratio of infant to under 5 y mortality was 62/102 per 1,000 live births (15), in Adamawa, Borno, and Yobe surveys suggested a lower proportion of infants among under 5 y deaths (*Discussion*). The crude birth rate was slightly lower than nationally (38 per 1,000 person-years), and surveys mostly reported negative net household migration (more people migrating out of than into households). Generally, the most extreme estimates were recorded in late 2016 to middle 2017 (Fig. 1).

**Table 1.** Characteristics of analysis-eligible ground mortality surveys

	Overall	2016	2017	2018
Eligible surveys (N)	70	13	31	26
CDR (per 10,000 person-days)	0.55 (0.17 to 1.58)	0.48 (0.25 to 1.58)	0.83 (0.29 to 1.57)	0.46 (0.17 to 1.06)
U5DR (per 10,000 child-days)	1.14 (0.23 to 4.46)	0.94 (0.53 to 1.90)	1.75 (0.56 to 4.46)	0.79 (0.23 to 2.25)
Proportion of <5 yo deaths that were among infants <1 yo	0.35 (0.00 to 1.00)	0.29 (0.00 to 0.60)	0.35 (0.00 to 0.80)	0.38 (0.00 to 1.00)
Household size (n)	5.3 (3.8 to 7.3)	5.3 (3.8 to 6.0)	5.3 (4.3 to 6.3)	5.3 (4.4 to 7.3)
Proportion of children aged under 5 y	0.19 (0.14 to 0.29)	0.18 (0.14 to 0.29)	0.19 (0.16 to 0.26)	0.18 (0.16 to 0.22)
Proportion of females in household	0.50 (0.47 to 0.60)	0.51 (0.50 to 0.60)	0.50 (0.47 to 0.53)	0.50 (0.48 to 0.52)
Crude birth rate (per 1,000 person-years)	33.7 (7.3 to 90.3)	37.4 (17.9 to 44.6)	41.7 (14.2 to 82.7)	23.7 (7.3 to 90.3)
Net migration rate (per 1,000 person-years)	−42.0 (−119.8 to 133.3)	−8.1 (−52.3 to 133.3)	−63.2 (−119.8 to 0.0)	−48.7 (−89.9 to −5.2)
Injury-specific death rate^*^ (per 10,000 person-days)	0.16 (0.00 to 1.44)	1.07 (0.28 to 1.44)	0.02 (0.00 to 0.78)	no data

^*^Based on 25 surveys that collected this information (5 in 2016, 20 in 2017 and none in 2018).

The median (interquartile range) of survey point estimates are reported unless noted.

### Predictive Model.

We fitted predictive statistical models for both CDR and U5DR, with four predictor variables (Table 2). Models indicated that CDR was higher among agro-pastoralists and with increasing price of maize, one of the main cereal staples in northeast Nigeria; it decreased as humanitarian actor presence intensified, and with increasing vaccination geocoverage. For U5DR, the association with geocoverage was not evident. Model predictions at the LGA level were mostly well aligned with observations (*SI Appendix*, Figs. S11 and S12), with minor systematic underprediction (Table 2).

**Table 2.** Fixed-effect model coefficients and measures of predictive performance

	CDR		U5DR	
Fixed effect	Rate ratio (95% CI^*^)	*P*-value	Rate ratio (95% CI^*^)	*P*-value
Main livelihood type				
Pastoralists [ref.]			–	
Agro-pastoralists	1.20 (0.97 to 1.49)	0.098	1.29 (1.01 to 1.66)	0.043
Number of humanitarian actors per 100,000 population (lag = 3 mo)				
≤2.5 [ref.]	–		–	
2.5 to 4.9	0.99 (0.82 to 1.19)	0.905	0.95 (0.74 to 1.23)	0.702
5.0 to 7.4	0.87 (0.68 to 1.11)	0.257	0.99 (0.71 to 1.37)	0.954
≥7.5	0.61 (0.46 to 0.81)	<0.001	0.67 (0.48 to 0.94)	0.022
Wholesale price of 1 kg of white maize (USD, 2010) (lag = 3 mo)				
≤0.25 [ref.]	–		–	
0.25 to 0.34	1.50 (1.12 to 2.02)	0.007	1.65 (1.04 to 2.62)	0.032
≥0.35	2.29 (1.73 to 3.03)	<0.001	2.93 (1.91 to 4.50)	<0.001
Vaccination geocoverage				
≤25% [ref.]	–			
25 to 49%	1.03 (0.62 to 1.70)	0.917	1.35 (0.56 to 3.22)	0.500
50 to 74%	0.82 (0.49 to 1.36)	0.444	1.26 (0.53 to 3.00)	0.597
≥75%	0.50 (0.24 to 1.01)	0.052	0.68 (0.23 to 2.03)	0.486
Predictive performance (at the LGA level)				
Dawid-Sebastiani score (on cross-validation)	32.6 (214.9)		74.3 (134.4)	
Mean square error (on cross-validation)	51.3 (252.1)		15.8 (41.6)	
Relative bias	−8.7%		−7.1%	
Relative precision of 95% CIs^*^	±59.7%		±60.1%	

^*^CI = confidence interval.

### Estimated Mortality.

Over a 45-mo period from April 2016 to December 2019, we estimate that about 908,100 deaths occurred in the three states combined (41% among children under 5 y; Table 3). When subtracting from this death toll mortality under the counterfactual, noncrisis scenario considered most likely (*Materials and Methods*), the estimated excess death toll was 490,900 (47% being children under 5 y); however, in the reasonable worst-case counterfactual scenario (i.e., highest noncrisis death rate leading to the lowest excess), a far smaller crisis-attributable death toll (85,400) was projected.

**Table 3.** Estimates (95% CIs) of actual, counterfactual, and excess mortality, for all ages and children under 5 y old, by state and counterfactual scenario

		Counterfactual deaths			Excess deaths		
State	Total deaths	Most likely	Reasonable worst-case	Reasonable best-case	Most likely	Reasonable worst-case	Reasonable best-case
All ages							
Adamawa	307,900 (187,600 to 506,600)	111,000 (53,800 to 229,400)	277,000 (171,100 to 447,900)	110,900 (53,700 to 229,200)	196,900 (133,800 to 277,200)	197,000 (133,900 to 277,400)	30,900 (16,500 to 58,700)
Borno	325,200 (218,600 to 491,300)	207,600 (112,900 to 383,800)	357,100 (236,000 to 543,500)	147,900 (76,200 to 288,100)	116,400 (103,000 to 118,300)	177,300 (142,300 to 203,100)	−31,900 (−52,200 to −17,400)
Yobe	275,000 (190,000 to 401,300)	98,500 (52,000 to 186,600)	188,600 (124,700 to 285,800)	98,300 (51,800 to 186,400)	176,500 (138,000 to 214,700)	176,700 (138,200 to 214,900)	86,400 (65,200 to 115,500)
total	908,100 (596,200 to 1,399,300)	417,200 (218,700 to 799,800)	822,700 (531,900 to 1,277,200)	357,100 (181,800 to 703,800)	490,900 (377,500 to 599,500)	551,000 (414,400 to 695,500)	85,400 (64,300 to 122,100)
Children under 5 y							
Adamawa	129,700 (76,900 to 221,200)	36,300 (14,400 to 91,900)	115,300 (69,700 to 190,700)	36,300 (14,300 to 91,800)	93,300 (62,500 to 129,300)	93,400 (62,600 to 129,400)	14,400 (7,200 to 30,500)
Borno	127,900 (76,900 to 218,400)	71,400 (31,700 to 161,500)	137,300 (83,900 to 225,800)	47,200 (19,200 to 116,600)	56,200 (45,000 to 59,300)	80,700 (57,700 to 101,800)	−9,200 (−9,700 to −6,300)
Yobe	113,800 (71,900 to 182,500)	32,800 (13,500 to 79,900)	72,500 (42,600 to 123,600)	32,700 (13,500 to 79,400)	81,000 (58,400 to 102,600)	81,100 (58,400 to 103,000)	41,300 (29,300 to 58,900)
total	371,300 (225,700 to 622,100)	140,500 (59,700 to 333,300)	325,100 (196,200 to 540,100)	116,200 (47,000 to 287,800)	230,800 (166,000 to 288,800)	255,200 (178,700 to 334,200)	46,200 (29,500 to 81,900)

**Fig. 2.**
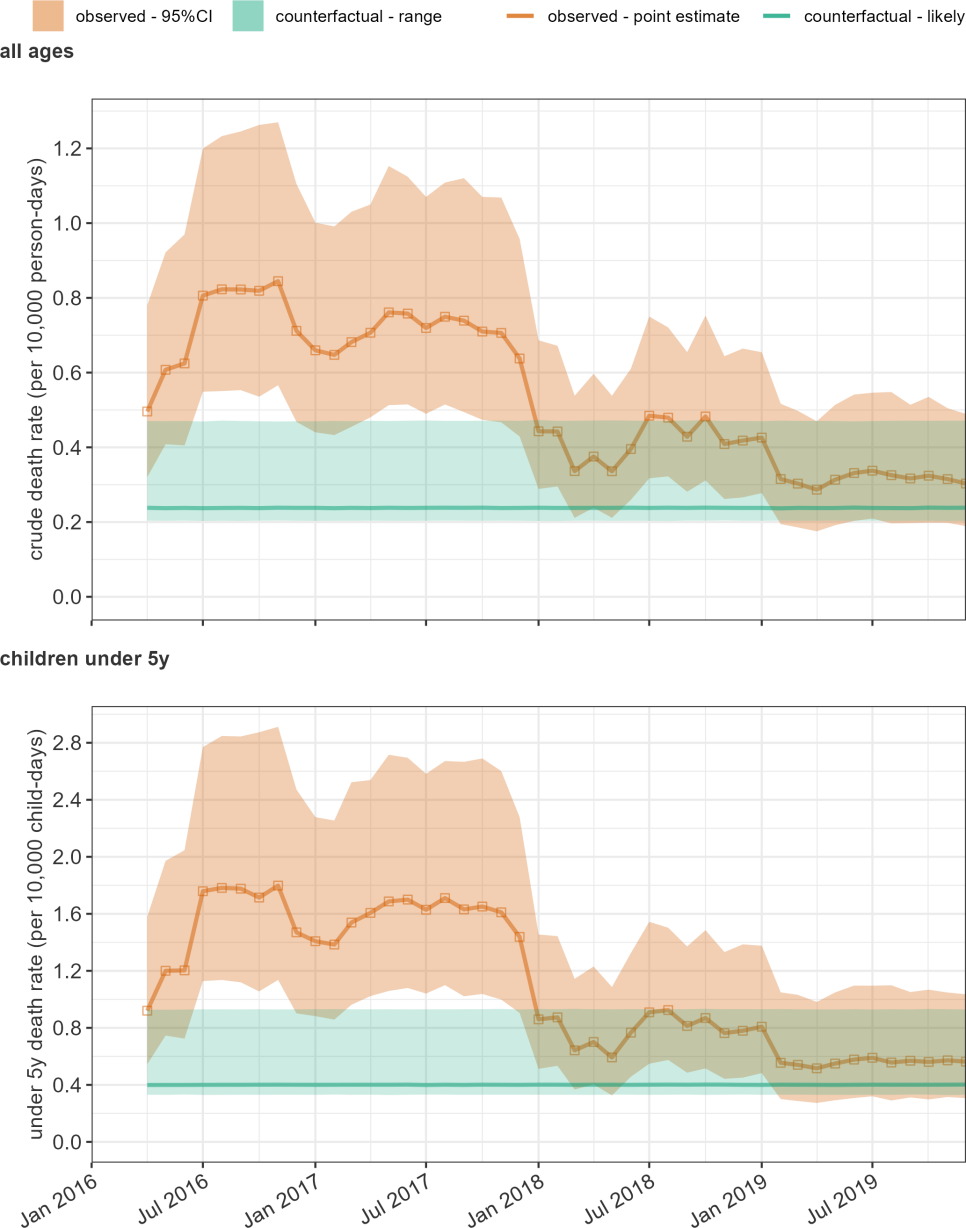
Trends in the estimated crude and U5DRs. The orange dotted line and shaded area indicate the point estimate and 95% CI under observed conditions. The green line indicates the most likely counterfactual level, and the green shaded area indicates the range between reasonable best- and worst-case counterfactual scenarios.

The highest death tolls occurred in 2016 and 2017, with more moderate levels in 2018 and 2019 (Table 4). CDR and U5DR estimates across the region displayed a similar trend (Fig. 2): CDR averaged 0.73 (95%CI 0.48 to 1.11) deaths per 10,000 person-days in 2016, 0.71 (0.48 to 1.06) in 2017, 0.42 (0.27 to 0.65) in 2018 and 0.32 (0.20 to 0.53) in 2019, with corresponding U5DR averages at 1.52 (0.95 to 2.48), 1.58 (1.00 to 2.54), 0.79 (0.47 to 1.36) and 0.58 (0.32 to 1.08). Overall, CDR and U5DR were between two and three times higher than the most likely counterfactual levels up to the end of 2018, and considerably higher than the projected CDR for Nigeria as a whole (0.34). Note that the differences between actual and counterfactual death rates are not linearly correlated to the differences in death tolls, as counterfactual scenarios also vary the extent of displacement and thus the denominators at risk across different LGAs.

**Table 4.** Estimates (95% CIs) of total and excess mortality, for all ages and children under 5 y old, by year

	All ages		Children under 5 y	
Year	Total deaths	Excess deaths	Total deaths	Excess deaths
2016^*^	239,700 (159,300 to 365,200)	160,000 (117,600 to 212,600)	99,700 (62,200 to 163,300)	72,900 (50,800 to 99,600)
2017	317,800 (213,900 to 477,800)	208,900 (156,900 to 269,200)	142,500 (89,800 to 228,700)	105,800 (74,200 to 141,700)
2018	194,800 (126,400 to 303,200)	82,300 (67,400 to 88,200)	73,500 (43,500 to 126,300)	35,400 (27,200 to 37,900)
2019	155,800 (96,600 to 253,100)	38,900 (29,900 to 39,600)	55,600 (30,300 to 103,800)	16,000 (11,000 to 16,500)

^*^Starting from April 2016, i.e., only 9 mo.

Only results for the most likely counterfactual scenario are shown.

**Fig. 3.**
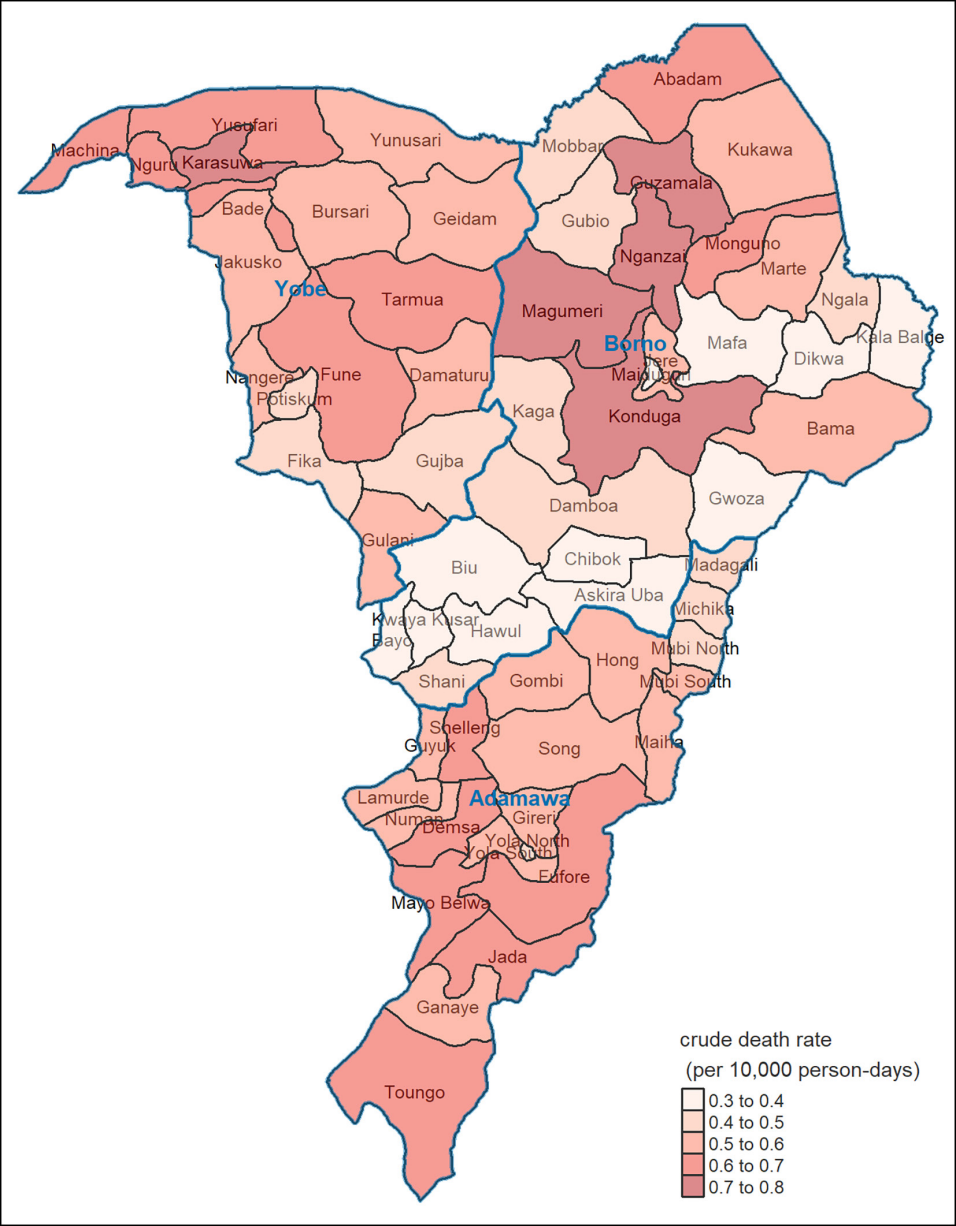
Estimated average CDR during the analysis period, by LGA.

Geographically, the highest, but also lowest, CDRs were estimated for LGAs in Borno state (Fig. 3). Notably, we projected consistently elevated CDR (compared to the counterfactual or, indeed, national estimates) across Adamawa and Yobe states. Note however that at the LGA level estimates are subject to considerable imprecision; furthermore, chronological trends are obfuscated by these period averages.

## Discussion

Our analysis finds that a crisis fueled by armed conflict likely caused a very large death toll over 4 y in Adamawa, Borno, and Yobe states, with about half of the excess mortality among children under 5 y old. Our central estimate suggests that between 2016 and 2019, the region lost about 4 to 5% of its population because of the crisis. Death rates were highest in 2016 to 2017 and reduced to less acute levels by 2019. Despite armed conflict and displacement being focalized in Borno state, we project equally or higher death tolls in Adamawa and Yobe (*Limitations*). Generally, estimates are subject to considerable uncertainty, particularly as the counterfactual assumptions are varied. Counterfactuals aside, however, the estimated CDR was about double the UN projections for Nigeria as a whole, at least before 2019: this observation alone provides a stark illustration of the health status differential between this crisis region and more stable Nigerian communities. Our estimate is temporally and geographically incomplete, as it omits a long period (2009 to 2015) of conflict between Nigerian authorities and militant groups, and, in particular, the 2013 to 2015 escalation period during which large waves of displacement occurred and humanitarian assistance was scarce. It also does not cover the experience of refugees and communities in Niger, Chad, and Cameroon affected by what has turned into a transnational conflict. The 2019 Nigeria Demographic and Health Survey provides state-specific estimates of child survival during 2011 to 2018 (17): in contrast to our study and source SMART (Standardized Monitoring and Assessment of Relief and Transition) surveys, this large-scale survey does not suggest a marked elevation in under 5 y mortality in Adamawa (104 per 1,000 live births), Borno (84) or Yobe (152), compared to the countrywide average (132). However, for Borno the survey replaced 39% of sampled clusters due to insecurity, and estimates are not disaggregated for the acute crisis period of 2015 to 2017.

Over the past decades, few attempts have been made to document mortality at the level of entire crises. However, studies have consistently illustrated the stark impact of armed conflict, displacement, and/or food insecurity on human survival. In the Democratic Republic of Congo, successive nationwide surveys estimated CDRs 1.4 to 1.7 times higher than prewar, with >4M excess deaths between 1998 and 2006 (18, 19). Surveys in Iraq between 2004 and 2011 found death tolls in the hundreds of thousands, with CDR about 1.5 to 3 times preinvasion levels (20), and similar elevations have been noted more recently in the Central African Republic (21). We have used successive iterations of the small-area estimation method in different settings. During a famine in Somalia (2010 to 2012), the method found that CDR increased about fivefold to eightfold, with some 260,000 excess deaths (22), while in South Sudan we estimated 380,000 excess deaths, about 1.7 times the counterfactual (23). Repeat Somalia studies suggested about 45,000 excess deaths during the 2016 to 2018 drought (24) and a similar amount during the first year of the ongoing drought (25).

Generally, proximity to armed conflict is associated with reduced child survival and increased maternal mortality, and as of 2017, some 265 M women and 368 M children were living dangerously near conflict areas, with conflict intensity also displaying a dose–response relationship with mortality (26, 27). Death rates appear systematically higher among IDPs than nondisplaced or refugee populations (16). The relative contribution of specific risk factors to conflict mortality is not systematically documented (27). While in high-intensity conflicts (e.g., Syria, Ukraine, Iraq) it is plausible that war injuries have driven excess mortality, we speculate that, in less active conflict scenarios within settings characterized by infections and malnutrition as the main contributors to disease burden, most excess deaths are indirectly attributable to conflict and caused by food insecurity, displacement, epidemics, and service disruptions as downstream consequences of armed conflict.

We have previously reviewed the relative benefits of alternative crisis mortality estimation methods and the potential applicability of small-area estimation (13, 28). Generally, prospective surveillance studies may generate the most immediately actionable information, but these require sustained efforts, access, and meaningful benefits for communities. Surveys also rely on unfettered access and ground authorizations, but can, if done at scale and with careful control of response and sampling biases, capture multiyear retrospective trends. Our statistical approach makes efficient use of existing information, features quantifiable validity, and reconstructs patterns for different periods or geographical strata, even where no data collection was done. It requires, however, access to a critical mass of mortality and predictor ground data. Predictors need to refer to a causal framework so as to capture variables that are sensitive to crisis conditions and thus can be varied to construct counterfactual scenarios; however, the causal framework needs to be context-specific (accordingly, a crisis resulting mainly from drought or economic decline, rather than insecurity, would probably warrant focus on climate, economic, and service performance variables). More recently, we have tested alternatives including analysis of satellite images of cemeteries (29, 30), capture-recapture analysis of key informant lists (31), and surveys of diaspora members, but these methods have so far been used in specific sites or to capture patterns rather than overall death tolls.

### Limitations.

Our statistical model for CDR appears consistent with the observed data and, importantly, features plausible, dose–response associations. The model for U5DR is less accurate, presumably reflecting sparser input data. The overall estimates rest on strong assumptions that any bias in the input data themselves does not have a substantial influence on the model and its predictions. We did sensitivity analyses to explore two key sources of potential bias: i) inaccurate estimation of IDP and population numbers and ii) underascertainment of under 5 y deaths. First, we assumed various levels of under- or overestimation in IDP figures (from 0.5 to 1.5 as a ratio of actual to observed figures) and population estimates (from 0.7 to 1.3), repeating all downstream analysis steps with the resulting alternative population denominators. Results appear highly sensitive to bias in population figures, but far less to IDP data bias (*SI Appendix*, Fig. S13). Second, we assumed that a proportion up to 50% of under 5 y deaths (neonates and infants in particular) were missed during ground survey household interviews: this is a potential limitation of SMART surveys (32), particularly where questionnaires are administered suboptimally or child deaths are stigmatizing, and may underlie the relatively low proportion of infants among all under 5 y deaths within the surveys we analyzed. Death tolls among children under 5 y would approximately double if only half of childhood deaths had been detected (*SI Appendix*, Fig. S14).

**Fig. 4.**
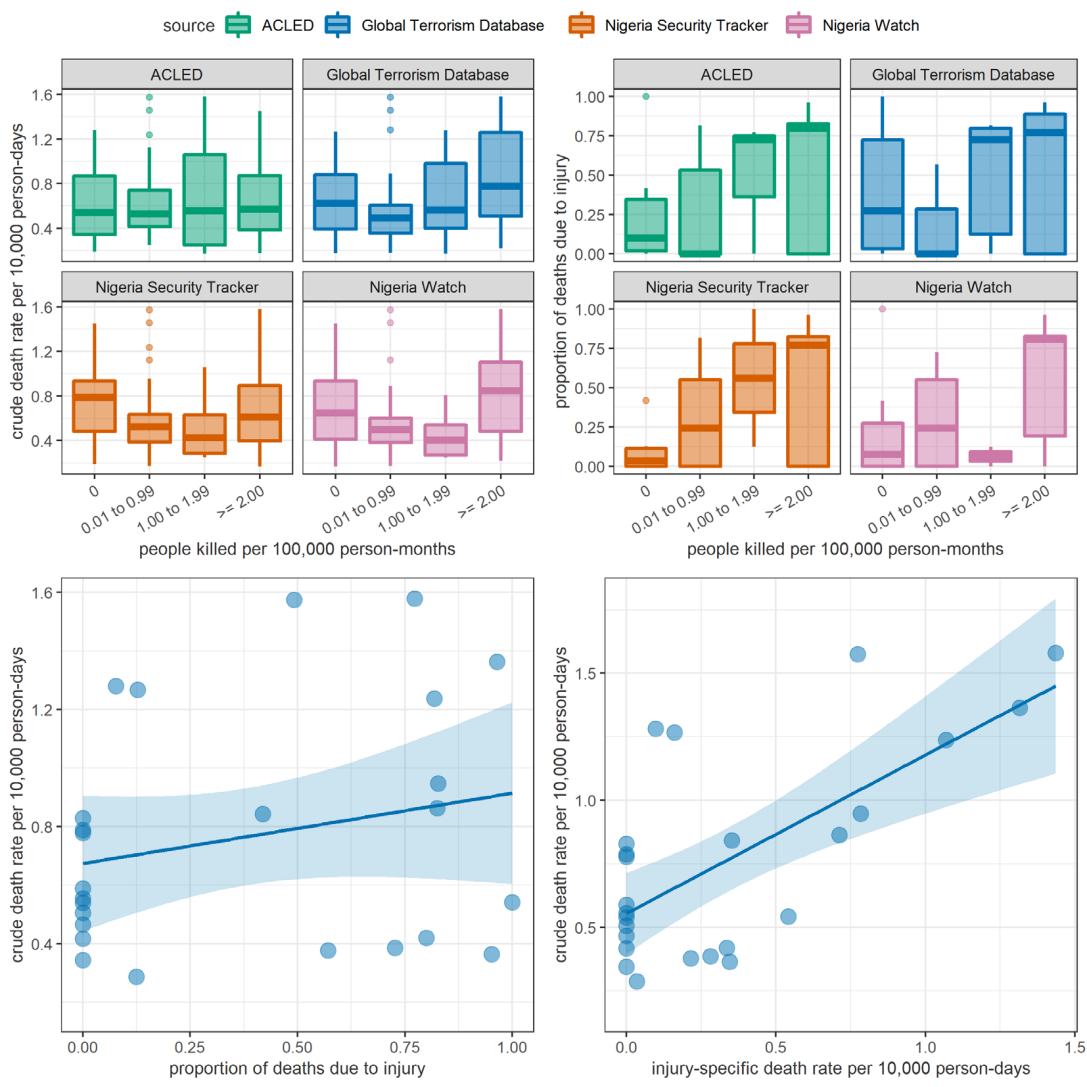
Correlations among different mortality indicators as estimated by ground SMART surveys and the rate of people killed as reported by alternative insecurity monitoring projects. In the top panels, box plots indicate the median and interquartile range; whiskers indicate the 95% percentiles. In the bottom panels, straight lines and shaded areas indicate linear regression best fits and 95% CIs, as a crude representation of the extent of correlation. ACLED = Armed Conflict Location & Event Data Project.

Our study does not shed light on cause-specific mortality. A countrywide verbal autopsy study, concurrent to ours, suggested that causes of death among children under 5 y in northeast Nigeria during 2013 to 2018 were broadly similar to the countrywide picture, with sepsis, intrapartum injury, malaria, diarrhea, and pneumonia as the leading causes (33). Although 25 of the ground surveys we analyzed collected cause-of-death information from next-of-kin, these surveys were clustered in time and space and were thus not considered a sufficient training and validation dataset for statistical models. However, the very large proportion of injury deaths, particularly in Borno and during 2016, combined with the strikingly higher risk of dying among males than among females, are consistent with violence having been a leading direct cause of mortality. As part of identifying potential model predictors, we sourced four different databases of insecurity collected by dedicated projects: these databases tallied 10,944 (Armed Conflict Location and Event Data Project), 10,540 (Nigeria Security Tracker), 4,859 (Global Terrorism Database), and 13,300 (Nigeria Watch) people killed during our same analysis period (note that these totals reflect not just sensitivity of detection, but also database-specific inclusion criteria). Unexpectedly, none of these sources of data, despite well-documented and systematic procedures for data collection and curation, were correlated with CDR or U5DR in our models, in contrast with previous analyses in Somalia (22, 23) and South Sudan (23), where insecurity events were a key overall mortality predictor, and with global analyses of the association of armed conflict intensity with child mortality (26). As shown in Fig. 4, while the rate of killings reported by different sources during the recall period of SMART surveys does broadly correlate positively with the proportion of deaths reported in the surveys as injury-related (top-right panel), there is no visible correlation of the rate of killings with CDR itself (top-left). Similarly, where surveys did collect information on cause of death, a correlation between CDR and the injury-specific death rate is visible (bottom-right), but not between CDR and proportion of injury deaths. One explanation for this lack of association could be that insecurity monitoring projects face a common challenge of ascertaining events and deaths in inaccessible areas, and generally where overall mortality is highest; this seems unlikely, as in fact all four sources do show much higher incidence of killings in partially or completely inaccessible LGAs, albeit with somewhat discordant patterns (*SI Appendix*, Fig. S15). We speculate that the restriction of surveys’ effective sampling frame to accessible, relatively secure areas of LGAs may provide some explanation for these counterintuitive patterns, by effectively decoupling the level of insecurity from survey-estimated death rates.

The above in turn raises the possibility of bias in our estimates due to inaccessible areas being systematically excluded from ground surveys and measurement of predictor data. We explored this in sensitivity analysis by assuming that in partially or completely inaccessible LGA-months the true CDR and U5DR were up to twice our estimates. Estimates were most sensitive to potential underestimation in Borno state, where access was most constrained (*SI Appendix*, Fig. S16); overall, the median estimate of excess death toll increased from 490,000 to 800,000 in an extreme bias scenario (doubling of CDR). We consider it plausible that, at least for Borno state, our study does substantially underestimate mortality.

Despite the very sizeable internal displacement during the analysis period, we also did not find that displacement indicators (proportion of IDPs, arrival or departure rate) were associated with mortality, though globally IDPs consistently have higher death rates than resident populations (16). Locations with very large IDP concentrations would have been relatively more secure (e.g., Maiduguri and Jere LGAs), and benefited disproportionately from humanitarian assistance or the generosity of local civil society; moreover, during this crisis, IDPs have preferentially settled among resident populations or in small camps, avoiding some of the public health threats of large, unplanned camps with no nearby essential services.

More fundamentally, our method requires deliberate definitions of what a crisis consists of, and of counterfactual conditions in its absence. These conditions are in turn set by assuming values of model predictors (and of demographic data) in the absence of the crisis. Specifically, we considered inflation among the conditions contributing to the crisis: indeed, increasing price of cereal, as a proxy of this condition, strongly predicted mortality. As shown in the *SI Appendix*, Fig. S9, the period of high prices in 2016 to 2017 was a nationwide phenomenon, and thus arguably not a specific feature of the north-east Nigeria crisis. However, for our central counterfactual scenario, we reasoned that in the crisis-affected region increased food prices would have had a disproportionate impact on risk of death given the depletion of coping mechanisms, reduced household budgets for nonfood items, low access to public health services and displacement conditions not encountered by other Nigerian populations. Our most likely mortality estimates thus reflect the added effect of food price increases; our reasonable-best scenario estimate, by contrast, assumes moderately high food prices as part of the expected baseline.

Last, our study was conducted mostly from Europe, with limited field presence and insight into the north-east Nigerian context. It would have been more appropriate to more closely involve Nigerian academic or civil society partners in its conduct and interpretation. For example, this lack of insight could have blinded us to validity problems with the datasets we relied on or misconceptions in the counterfactual scenarios we constructed. As part of ongoing Somalia-focused estimation, we are transferring ownership of methods to a Somali university: this should be a deliberate objective of all similar analyses and warrants donor support.

### Conclusions

Estimates of the direct and indirect mortality impact of armed conflict and other crisis conditions are needed to appraise the appropriateness of humanitarian responses, but also for governments, combatants, and societies to appreciate the consequences of these crises and of military tactics used to prosecute wars. More fundamentally, they help to memorialize those who died and construct objective historical accounts. Our attempt at deriving such an estimate for north-east Nigeria should be critiqued and interpreted in light of its limitations. We contend, however, that generating evidence on the human toll of this very large crisis warrants the involvement of several independent groups, perhaps leveraging different methods. Generally, mortality estimation should become a predictable, systematic public health information service across all crisis settings. This requires concerted work by scientific groups and sustained, long-term funding support.

## Materials and Methods

### Overview of the Approach.

We used a small-area estimation (34) statistical modeling approach relying on datasets collected by various organizations as part of the humanitarian response in Northeast Nigeria, and not requiring primary data collection. The method consists of six steps, extensively described in a separate paper (28) and enables estimation by LGA (equivalent to a district or county) and month. Statistical code and a minimal dataset to implement the analysis using R software (35) are available on https://github.com/francescochecchi/mortality_small_area_estimation/tree/nga: These allow users to vary various data management and modeling parameters by modifying their values on Microsoft Excel input files.

Briefly, the sequential steps comprise of 1) identifying, managing, and grading the quality of existing population mortality surveys done in the region and time period of interest (these surveys provide the data on which the model is trained); 2) reconstructing population denominators for each LGA-month by applying assumed growth rates to existing alternative point-in-time population source, while also adjusting for internal and refugee displacement in and out of each LGA and grading the quality of each alternative source to compute a weighted mean (population denominators are used to convert between death rates and tolls and to calculate per capita quantities of model predictors); 3) identifying datasets that offer proxy or direct measures of various potential factors along a causal pathway leading to mortality, and preparing these potential “predictor” datasets for analysis by imputing missing values, smoothing or interpolation, creating lags, etc.; 4) combining ground mortality, population and predictor data into a statistical model, selected on the basis of predictive accuracy on cross-validation (i.e. on a simulated external dataset), the plausibility of observed associations and the sensitivity of predictor variables to crisis conditions; 5) using the model to predict mortality (both death toll and rate) for all LGA-months, i) as per the observed data values and ii) per counterfactual scenarios defined by assumptions about which values the model predictors and population denominators might have taken in the absence of a crisis—here, the difference between observed-conditions and counterfactual predictions provides the estimated excess mortality; and 6) performing specific sensitivity analyses, e.g. to explore the effect of potential bias in input data. We implement steps (4) to (6) for both the CDR (deaths due to all causes among all ages per person-time) and the U5DR (deaths due to all causes among children under 5 y per child-time). Step (2) does not depend on the other steps and its methods are specific to the Nigeria context: for brevity’s sake this step is summarized in *SI Appendix*. Reconstructed population denominators are included as a Dataset.

### Study Population and Timeframe.

We collected data up to December 2019; we restricted the analysis period to 2016 to 2019 due to data sparsity pre-2016 and the unclear effect of the COVID-19 pandemic in 2020. However, pre-2016 values for predictor variables, where available, were used to inform counterfactual scenarios. Adamawa, Borno, and Yobe states are divided into 21, 27, and 17 LGAs respectively, and, as of July 2017 (the mid-point of our analysis period), had populations of 3.9 (8% internally displaced), 5.0 (29%), and 3.4 (11%) million, namely 12.3 million overall (18%), according to our reconstruction (*SI Appendix*).

### Data Sources.

#### Ground mortality data.

We accessed reports and raw datasets of 73 multistage cluster sampling household surveys conducted in the region during 2016 to 2018 by either the National Bureau of Statistics [NBS, supported by the United Nations Children's Fund (UNICEF) and the United States Centers for Disease Control and Prevention, some of which previously published (36); N = 57], UNICEF (N = 7) or Action Against Hunger (N = 9), all using the standardized methods, survey instruments, and analytic tools of the SMART project (37). SMART surveys are routinely conducted across crisis settings; while primarily focusing on nutritional status, they usually feature a mortality questionnaire module that elicits information from respondents on the size and evolution (births, deaths, arrivals, and departures) of their nuclear (i.e. regularly sharing meals) household members over a retrospective “recall” period, enabling estimation of various demographic quantities including the CDR and U5DR (38). The sampling universe of NBS surveys was a “domain” (grouping of LGAs: *SI Appendix*), but raw datasets contained information on which LGA each survey cluster fell within; remaining surveys were conducted with single LGAs as their sampling universe. Surveys were implemented at regular intervals with a median recall period of 135 d (range 90 to 309): The combination of frequency and geographic breadth of data collection yielded high person-time survey coverage (*SI Appendix*). We excluded two surveys due to incomplete datasets and one due to highly unusual estimates. For each of the remaining 70, we reanalyzed raw datasets, extracted quality scores (*SI Appendix*) and reviewed the report to quantify possible selection bias. Individual survey characteristics and their person-time coverage are detailed in *SI Appendix*, Table S2 and Fig. S2 respectively. Most (59/70) surveys covered ≥75% of their target sampling universe.

#### Predictor data.

Guided by an a priori causal framework (12), we identified candidate datasets from which to attain information on predictor variables (Table 5) through thorough internet searches and meetings with governmental agencies and humanitarian coordination mechanisms in Maiduguri and Abuja, during which the study was socialized and various possible data sources were reviewed for their suitability. Details on data management are in *SI Appendix*. Several potentially relevant predictor datasets had incomplete geographic (e.g. only Borno state) or time period coverage, or were too sparse to support imputation, and were thus excluded from analysis: these included tracking of attacks against aid workers; settlement intactness and habitation data collected by the polio eradication programme; Emergency Food Security Assessments done at LGA, domain or state level once or twice a year; health facility functionality data collected through the Health Resources Availability Monitoring System by the World Health Organization; epidemic surveillance data from WHO’s Early Warning, Alert and Response System in Borno; and measles incidence data from Yobe’s State Ministry of Health.

**Table 5.** Candidate predictor datasets used to build the model

Variable	Value(s)	Source and URL if public	Notes and assumptions
Climate			
Rainfall	Mean of standard precipitation index (number of SDs away from the historical average rainfall during the same period of the year)	Climate Engine (UCSB-CHG/CHIRPS/PENTAD dataset downloadable by logging into https://app.climateengine.org/login; more documentation on https://docs.climateengine.org/docs/build/html/index.html)	Used 5-d Climate Hazards Group InfraRed Precipitation with Station data dataset (39), with historical data starting in 1981. Explored lags of 0 to 6 mo and 2 to 3 mo running means.
Season	Whether a given month is in the lean season or harvest season	Famine early warning systems network http://fews.net/file/87830	As per expectations of a typical year.
Insecurity			
Incidence of insecurity events^*^	events per 100,000 population	Armed Conflict Location & Event Data Project (ACLED) (40) https://acleddata.com	Retained all ACLED event types. Explored lags of 0 to 4 mo.
Rate of people killed^*^	deaths per 100,000 population
Rate of people killed^*^	deaths per 100,000 population	Nigeria security tracker, Council on foreign relations https://www.cfr.org/nigeria/nigeria-security-tracker/p29483	Explored lags of 0 to 4 mo.
Incidence of insecurity events^*^	events per 100,000 population	Global terrorism database, University of Maryland https://www.start.umd.edu/gtd/	The database is restricted to violence by nonstate actors. We used reported GPS coordinates to identify the LGA. Explored lags of 0 to 4 mo.
Rate of people killed^*^	deaths per 100,000 population
Incidence of insecurity events^*^	events per 100,000 population	Nigeria watch, Institut de Recherche pour le Développement http://www.nigeriawatch.org/ (available at a fee)	Included all events tagged as “political issue”, “religious issue”, “land issue”, “market issue” and “cattle grazing”. Explored lags of 0 to 4 mo.
Rate of people killed^*^	deaths per 100,000 population		
Displacement			
Proportion of IDPs^*^	IDPs living within the LGA, out of LGA population	As per our population estimation.		
IDP departure rate^*^	IDPs leaving the LGA per 100,000	As per our population estimation.		
IDP arrival rate^*^	IDPs arriving into the LGA per 100,000	As per our population estimation.	
Food security and livelihoods
Main local livelihood type	Pastoralists, agro-pastoralists	Famine early warning systems network http://fews.net/west-africa/nigeria/livelihood-zone-map/february-2019	Grouped 11 livelihood zones into two categories. Assumed to be static.
Market prices	Price of 1 kg of staple cereals (wholesale or retail) Price of 1 L of gasoline fuel Price of 1 loaf of bread	World food programme https://data.humdata.org/dataset/wfp-food-prices-for-nigeria	Explored lags of 0 to 3 mo. See *SI Appendix* for further notes on data management.
Humanitarian (public health) services
Humanitarian actor presence^*^	Number of humanitarian actors present per 100,000 population (all sectors; public health only = health, nutrition and water, hygiene & sanitation)	United Nations Office for coordination of humanitarian affairs https://reliefweb.int/updates?advanced-search=%28C175%29_%28T4590%29_%28F12570%29	Explored lags of 0 to 3 mo.
Accessibility by humanitarian actors	Partial/none, Full	Reconstructed by the authors based on systematic review of UN infographics and maps https://reliefweb.int/updates?view=maps&advanced-search=%28C175%29_%28F12570%29	
Incidence of admissions for severe acute malnutrition^*^	Number of children admitted to therapeutic feeding services per 100,000 population	Nutrition Cluster Information Working Group, Abuja/Maiduguri (not public)	Proxy for nutritional service coverage. Also a proxy for nutritional status.
Quality of case management of severe acute malnutrition	Proportion of children admitted who are discharged successfully (survive and are not lost to follow-up or transferred)	Nutrition Cluster Information Working Group, Abuja/Maiduguri (not public)	Proxy for service quality.
Vaccination geocoverage		Nigeria vaccination tracking system http://vts.eocng.org/ChronicalCoverage/Index (website no longer accessible) (41–43)	Proxy for overall health service availability and coverage. See *SI Appendix* for further notes on data management.

^*^Divided by district population estimates to obtain a population rate.

With the exception of market prices (*SI Appendix*), all variables were available at the LGA-month level of stratification.

### Analysis.

#### Predictive model.

Given the data structure (household-level mortality observations, LGA-level predictor data, and multiple rounds of mortality surveys for each LGA, with de novo samples each time), we fitted a mixed quasi-Poisson model to the count of deaths within each household, offset by the natural log of household person-time at risk during the survey recall period, with a random effect for LGA and weights for survey robustness scores (range 0 to 1, and equal to the product of survey quality score and sampling coverage, thereby reducing the influence of surveys with low quality and/or potential for selection bias; see *SI Appendix*). We calculated mean monthly values of analysis-eligible predictors over the recall period of each mortality survey. For both CDR and U5DR, we selected predictors by i) searching across all plausible lags and continuous versus categorical versions of each variable; ii) combining the most data-consistent (lowest F-test *P*-value) among these variants into all possible multivariable models, and shortlisting models based on predictive accuracy [low Dawid–Sebastiani scoring rule (44)]; iii) selecting a single fixed-effects model from the shortlist based on its performance on 10-fold LGA-level cross-validation and the plausibility of observed associations; iv) testing for plausible interactions; and v) adding the random effect, retaining the mixed model if it offered superior predictive accuracy on cross-validation. We used the glmmTMB package for mixed modeling (45).

#### Mortality estimation.

Excess mortality may be defined as the difference between deaths under observed and counterfactual conditions, the latter constituting expected levels in the absence of a crisis. We thus defined most likely (i.e., average), reasonable best- and reasonable worst-case counterfactual scenarios for predictors and population denominators (Table 6). The most likely scenario attempts to model what would plausibly have happened in 2016 to 2019 if there had been no price inflation, if humanitarian presence had not been scaled up from 2016 onwards, if vaccination coverage had been similar to the rest of Nigeria and if displacement due to the conflict had not occurred. We used the model to predict mortality given the observed and counterfactual data, and the resulting excess. Specifically, for both observed and counterfactual conditions, for both CDR and U5DR and for each LGA-month we drew 1,000 bootstrap random values with replacement from the normal distributions arising from corresponding models’ log estimates and SEs. After multiplying these bootstrap samples by population denominators, we took the median, 2.5th and 97.5th percentiles of resulting death tolls as the point estimate and CI, which we then aggregated by year, state, and overall. As such, estimates propagate model error, but no other source of uncertainty (see *Discussion* and sensitivity analyses in *SI Appendix*).

**Table 6.** Counterfactual value assumptions

Variable	Scenario	Value	Justification
Predictors			
Price of white maize	most likely	Mean of values in Jan-Jun 2015 and 2018 to 2020, for each LGA	Available periods before and after the price inflation period, as determined through visual inspection (*SI Appendix*).
	reasonable worst-case	Mean of entire 2015 to 2020 series for each LGA	Assumes that periods of price inflation do not constitute an unusual crisis, but rather are part of normal fluctuation in prices.
	reasonable best-case	Minimum of entire 2015 to 2020 series for each LGA	Most optimistic assumption given available data.
Presence of humanitarian actors	most likely	Baseline at the start (generally very limited humanitarian presence)	It is unlikely that humanitarian actors would have expanded their presence in the absence of armed conflict.
	reasonable worst-case	No humanitarian assistance whatsoever	
	reasonable best-case	Baseline at the start (generally very limited humanitarian presence)	As above.
Vaccination geocoverage	most likely	Point estimate of coverage in non-BAY^*^ states, by month in 2016 to 2019	In the absence of armed conflict, the affected region would have achieved a coverage no worse than elsewhere in Nigeria. See *SI Appendix* for data sources.
	reasonable worst-case	lower 95% CI of coverage in non-BAY states, by month in 2016 to 2019	
	reasonable best-case	upper 95% CI of coverage in non-BAY states, by month in 2016 to 2019	
Population denominators			
Number of IDPs	most likely	average proportion of displacement not due to the insurgency [=5%, from International Organisation for Migration (IOM) dataset]	Some displacement did occur specifically due to natural disasters.
	reasonable worst-case	20% of the actual level	
	reasonable best-case	no displacement	
Number of refugees	most likely	average proportion of displacement not due to the insurgency (=5%, from IOM dataset)	As above.
	reasonable worst-case	20% of the actual level	
	reasonable best-case	no displacement	

^*^BAY = Borno, Adamawa, Yobe.

### Ethics.

All data were previously collected for routine humanitarian response and/or health service provision purposes and were either in the public domain or shared in fully anonymized format. The study was approved by the Ethics Committee of the London School of Hygiene & Tropical Medicine (ref. 15334) and the Nigerian Institute of Medical Research Institutional Review Board (ref. IRB/18/065).

### Disclaimer.

Geographical names and boundaries presented in this report are used solely for the purpose of producing scientific estimates and do not necessarily represent the views or official positions of the authors, the London School of Hygiene and Tropical Medicine, any of the agencies that have supplied data for this analysis, or the donor. The authors are solely responsible for the analyses presented here, and acknowledgment of data sources does not imply that the agencies or individuals providing data endorse the results of the analysis.

## Supplementary Material

Appendix 01 (PDF)

Dataset S01 (XLSX)

## Data Availability

Anonymised household-level survey datasets, aggregate, non-individual and fully anonymised data on morbidity, armed conflict intensity, humanitarian activity, market prices, climate and population denominators data have, as well as analysis code, have been deposited in GitHub (https://github.com/francescochecchi/mortality_small_area_estimation/tree/nga) (46). All study data are included in the article and/or supporting information.

## References

[1] F. C. Onuoha, “Why do youth join Boko Haram?” (Special Report 348: US Institute of Peace, Washington, DC, 2014). https://www.usip.org/publications/2014/06/why-do-youth-join-boko-haram. Accessed 14 April 2021.

[2] C. Kwaja, “Nigeria’s Pernicious drivers of ethno-religious conflict” (Africa Center for Strategic Studies, Washington, DC, 2011). https://africacenter.org/publication/nigerias-pernicious-drivers-of-ethno-religious-conflict/. Accessed 14 April 2021.

[3] A. Obe, “Environmental degradation, climate change and conflict: The Lake Chad basin area” (International Crisis Group, Lagos, Nigeria, 2016). https://medium.com/the-future-of-conflict/environmental-degradation-climate-change-and-conflict-the-lake-chad-basin-area-6aec2bd9fa25. Accessed 14 April 2021.

[4] “Nigeria: 2020 humanitarian needs overview” (United Nations Office for Coordination of Humanitarian Affairs, Abuja, Nigeria, 2019). https://reliefweb.int/report/nigeria/nigeria-humanitarian-needs-overview-2020-december-2019. Accessed 10 February 2022.

[5] J. Kurtzer, “Out of sight” (Center for Strategic and International Studies (CSIS), Washington, DC, 2020). https://www.csis.org/analysis/out-sight-northeast-nigerias-humanitarian-crisis. Accessed 10 February 2022.

[6] P. McIlreavy, J. Schopp, A collective shame: The response to the humanitarian crisis in north-eastern Nigeria. Humanitarian Practice Network **70**, 3 (2017).

[7] A. Stoddard, P. Harvey, M. Czwarno, M. Breckenridge, “Humanitarian access SCORE report: Northeast Nigeria. Survey on the coverage, operational reach, and effectiveness of humanitarian aid” (Humanitarian Outcomes, London, United Kingdom, 2020). https://www.humanitarianoutcomes.org/SCORE_report_NE_Nigeria_2020. Accessed 10 February 2022.

[8] “Food insecurity reaches extreme level in pockets of Nigeria’s Borno State” (Famine Early Warning Systems Network, Washington, DC, 2016). https://fews.net/west-africa/nigeria/alert/july-7-2016. Accessed 15 April 2021.

[9] “Cadre Harmonisé Update Analysis to Identify Risk Areas and Populations in Acute Food and Nutrition Insecurity in Adamawa, Borno and Yobe States of Nigeria” (Food and Agriculture Organization, Abuja, Nigeria, 2016). https://reliefweb.int/report/nigeria/cadre-harmonis-update-analysis-identify-risk-areas-and-populations-acute-food-and. Accessed 14 April 2021.

[10] “Special alert on Borno state, Nigeria: Urgent humanitarian action needed to respond to an elevated risk of famine” (Integrated Food Security Phase Classification, Abuja, Nigeria, 2016). https://reliefweb.int/report/nigeria/special-alert-borno-state-nigeria-urgent-humanitarian-action-needed-respond-elevated. Accessed 13 April 2021.

[11] O. Omole, H. Welye, S. Abimbola, Boko Haram insurgency: Implications for public health. Lancet **385**, 941 (2015). 10.1016/S0140-6736(15)60207-0 25747581

[12] F. Checchi , Public health information in crisis-affected populations: A review of methods and their use for advocacy and action. Lancet **390**, 2297–2313 (2017). 28602558 10.1016/S0140-6736(17)30702-X

[13] F. Checchi, “Estimation of population mortality in crisis-affected populations: Guidance for humanitarian coordination mechanisms” (World Health Organization, Geneva, 2018). https://healthcluster.who.int/publications/m/item/estimation-of-population-mortality-in-crisis-affected-populations. Accessed 12 March 2023.

[14] O. A. Makinde , Death registration in Nigeria: A systematic literature review of its performance and challenges. Glob. Health Action **13**, 1811476 (2020). 32892738 10.1080/16549716.2020.1811476 PMC7783065

[15] United Nations Department of Economic and Social Affairs, Population Division, “World population prospects 2019” (United Nations, New York, NY, 2019). https://population.un.org/wpp/. Accessed 10 March 2021.

[16] P. Heudtlass, N. Speybroeck, D. Guha-Sapir, Excess mortality in refugees, internally displaced persons and resident populations in complex humanitarian emergencies (1998–2012)–Insights from operational data. Confl. Health **10**, 15 (2016). 27441038 10.1186/s13031-016-0082-9 PMC4952240

[17] National Population Commission, ICF, “Nigeria demographic and health survey 2018–Final report” (National Population Commission of Nigeria, Abuja, Nigeria, 2019). https://dhsprogram.com/publications/publication-fr359-dhs-final-reports.cfm. Accessed 10 February 2023.

[18] B. Coghlan , Update on mortality in the Democratic Republic of Congo: Results from a third nationwide survey. Disaster Med. Public Health Prep. **3**, 88–96 (2009). 19491603 10.1097/DMP.0b013e3181a6e952

[19] B. Coghlan , Mortality in the Democratic Republic of Congo: A nationwide survey. Lancet **367**, 44–51 (2006). 16399152 10.1016/S0140-6736(06)67923-3

[20] A. Hagopian , Mortality in Iraq associated with the 2003–2011 war and occupation: Findings from a national cluster sample survey by the university collaborative Iraq mortality study. PLoS Med. **10**, e1001533 (2013). 24143140 10.1371/journal.pmed.1001533 PMC3797136

[21] K. B. A. Gang, J. O’Keeffe, Anonymous, L. Robert, Cross-sectional survey in Central African Republic finds mortality 4-times higher than UN statistics: How can we not know the Central African Republic is in such an acute humanitarian crisis? Confl. Health. **17**, 21 (2023). 37072800 10.1186/s13031-023-00514-z PMC10111645

[22] F. Checchi, W. Courtland Robinson, “Mortality among populations of southern and central Somalia affected by severe food insecurity and famine during 2010-2012” (Food and Agriculture Organization, Nairobi, Kenya, 2013). https://reliefweb.int/report/somalia/mortality-among-populations-southern-and-central-somalia-affected-severe-food. Accessed 10 February 2021.

[23] F. Checchi, A. Testa, A. Warsame, L. Quach, R. Burns, "Estimates of crisis-attributable mortality in South Sudan, December 2013- April 2018: A statistical analysis–South Sudan." (London School of Hygiene and Tropical Medicine, London, UK, 2018). https://reliefweb.int/report/south-sudan/estimates-crisis-attributable-mortality-south-sudan-december-2013-april-2018. Accessed 11 January 2021.

[24] A. Warsame, S. Frison, F. Checchi, Drought, armed conflict and population mortality in Somalia, 2014–2018: A statistical analysis. PLOS Glob. Public Health **3**, e0001136 (2023). 37043439 10.1371/journal.pgph.0001136 PMC10096495

[25] O. J. Watson, F. Checchi, "From insight to action: Examining mortality in Somalia" (London School of Hygiene and Tropical Medicine, London, UK, 2023). https://reliefweb.int/report/somalia/insight-action-examining-mortality-somalia. Accessed 20 April 2023.

[26] Z. Wagner , Armed conflict and child mortality in Africa: A geospatial analysis. Lancet **392**, 857–865 (2018). 30173907 10.1016/S0140-6736(18)31437-5 PMC6338336

[27] E. Bendavid , The effects of armed conflict on the health of women and children. Lancet **397**, 522–532 (2021). 33503456 10.1016/S0140-6736(21)00131-8 PMC7612212

[28] F. Checchi, A. Testa, A. Gimma, E. Koum-Besson, A. Warsame, A method for small-area estimation of population mortality in settings affected by crises. Popul. Health Metrics **20**, 4 (2022). 10.1186/s12963-022-00283-6 PMC8751462 35016675

[29] E. S. Koum Besson , Excess mortality during the COVID-19 pandemic: A geospatial and statistical analysis in Aden governorate, Yemen. BMJ Glob. Health **6**, e004564 (2021). 10.1136/bmjgh-2020-004564 PMC7992372 33758012

[30] A. Warsame , Excess mortality during the COVID-19 pandemic: A geospatial and statistical analysis in Mogadishu, Somalia. Intern. J. Infect. Dis. **113**, 190–199 (2021). 10.1016/j.ijid.2021.09.049 PMC8572680 34571148

[31] M. Alhaffar , Adult mortality before and during the first wave of COVID-19 pandemic in nine communities of Yemen: A key informant study. Confl. Health **16**, 63 (2022). 36510241 10.1186/s13031-022-00497-3 PMC9743127

[32] Working Group for Mortality Estimation in Emergencies, Wanted: Studies on mortality estimation methods for humanitarian emergencies, suggestions for future research. Emerg. Themes Epidemiol. **4**, 9 (2007). 17543103 10.1186/1742-7622-4-9 PMC1904216

[33] A. Odejimi , Causes of deaths in neonates and children aged 1–59 months in Nigeria: Verbal autopsy findings of 2019 Verbal and Social Autopsy study. BMC Public Health **22**, 1130 (2022). 35668378 10.1186/s12889-022-13507-z PMC9172014

[34] J. N. K. Rao, I. Molina, Small Area Estimation: Rao/Small Area Estimation (John Wiley & Sons, Inc, Hoboken, NJ, USA, 2015).

[35] R Core Team, R: A Language and Environment for Statistical Computing, version 4.1.0 (R Foundation for Statistical Computing, Vienna, Austria, 2021). https://www.R-project.org/

[36] E. Leidman , Acute malnutrition among children, mortality, and humanitarian interventions in conflict-affected regions— Nigeria, October 2016–March 2017. MMWR Morb. Mortal Wkly. Rep. **66**, 1332–1335 (2017). 29216027 10.15585/mmwr.mm6648a4 PMC5757638

[37] Standardised Monitoring and Assessment of Relief and Transitions (SMART), Measuring mortality, nutritional status, and food security in crisis situations: SMART methodology. https://smartmethodology.org/. Accessed 14 February 2021.

[38] K. L. Cairns , Cross-sectional survey methods to assess retrospectively mortality in humanitarian emergencies. Disasters **33**, 503–521 (2009). 19500327 10.1111/j.1467-7717.2008.01085.x

[39] C. Funk , The climate hazards infrared precipitation with stations—A new environmental record for monitoring extremes. Sci. Data **2**, 150066 (2015). 26646728 10.1038/sdata.2015.66 PMC4672685

[40] C. Raleigh, A. Linke, H. Hegre, J. Karlsen, Introducing ACLED: An armed conflict location and event dataset: Special data feature. J. Peace Res. **47**, 651–660 (2010).

[41] K. Touray , Tracking vaccination teams during polio campaigns in Northern Nigeria by use of geographic information system technology: 2013–2015. J. Infect Dis. **213**, S67–S72 (2016). 26609004 10.1093/infdis/jiv493 PMC4818548

[42] J. Higgins , Finding inhabited settlements and tracking vaccination progress: The application of satellite imagery analysis to guide the immunization response to confirmation of previously-undetected, ongoing endemic wild poliovirus transmission in Borno State, Nigeria. Int. J. Health Geogr. **18**, 11–11 (2019). 31096971 10.1186/s12942-019-0175-y PMC6524248

[43] I. Barau, M. Zubairu, M. N. Mwanza, V. Y. Seaman, Improving polio vaccination coverage in nigeria through the use of geographic information system technology. J. Infect. Dis. **210**, S102–S110 (2014). 25316823 10.1093/infdis/jiu010

[44] T. Gneiting, A. E. Raftery, Strictly proper scoring rules, prediction, and estimation. J. Am. Stat. Assoc. **102**, 359–378 (2007).

[45] M. E. Brooks , glmmTMB balances speed and flexibility among packages for zero-inflated generalized linear mixed modeling. R J. **9**, 378–400 (2017).

[46] F. Checchi, C. I. Jarvis, K. van Zandvoort, A. Warsame, The death toll of armed conflict and food insecurity in north-east Nigeria, 2016-2019: a statistical study - Explanation of R analysis scripts and input data files. Github. https://github.com/francescochecchi/mortality_small_area_estimation/tree/nga. Deposited April 2022.

