# Supplementary material for: Mortality among populations affected by armed conflict in northeast Nigeria, 2016 to 2019
Source: Proc Natl Acad Sci U S A. 2023 Jul 19;120(30):e2217601120. doi: 10.1073/pnas.2217601120 (PMC10372555; doi:10.1073/pnas.2217601120)
Supplement: Supplementary file 1 — Appendix 01 (PDF) [file pnas.2217601120.sapp.pdf]

Mortality among populations affected by armed conflict in northeast Nigeria, 2016 to 2019

SUPPORTING INFORMATION APPENDIX

Table of contents

Introduction 2
Trends in internal displacement and insecurity 2
Methods 3
Geographic strata 3
SMART mortality surveys 4
Reconstruction of population denominators 8
Completeness of predictor datasets 11
Management of predictor datasets 14
Market prices 14
Vaccination coverage 15
Results 17
Predictive model 17
Discussion 18
Sensitivity analysis: bias in displacement and population data 18
Sensitivity analysis: Under-estimation in U5DR 19
Trends in the rate of people killed, by accessibility status of LGAs 20
Sensitivity analysis: potential inaccessibility bias 21
References 22

Introduction

Trends in internal displacement and insecurity

Figure S1 shows trends in the number of new internally displaced persons (IDPs) due conflict across Nigeria, as well as the numbers of people killed in Adamawa, Borno and Yobe as reported by the four different insecurity data sources we used in our study. We could not identify any published data going back to at least 2009 on the number IDPs in the three states taken in isolation: as such, it is possible that the IDP trends graph is confounded by displacements due to other conflicts in Nigeria (e.g. northwest, southeast).

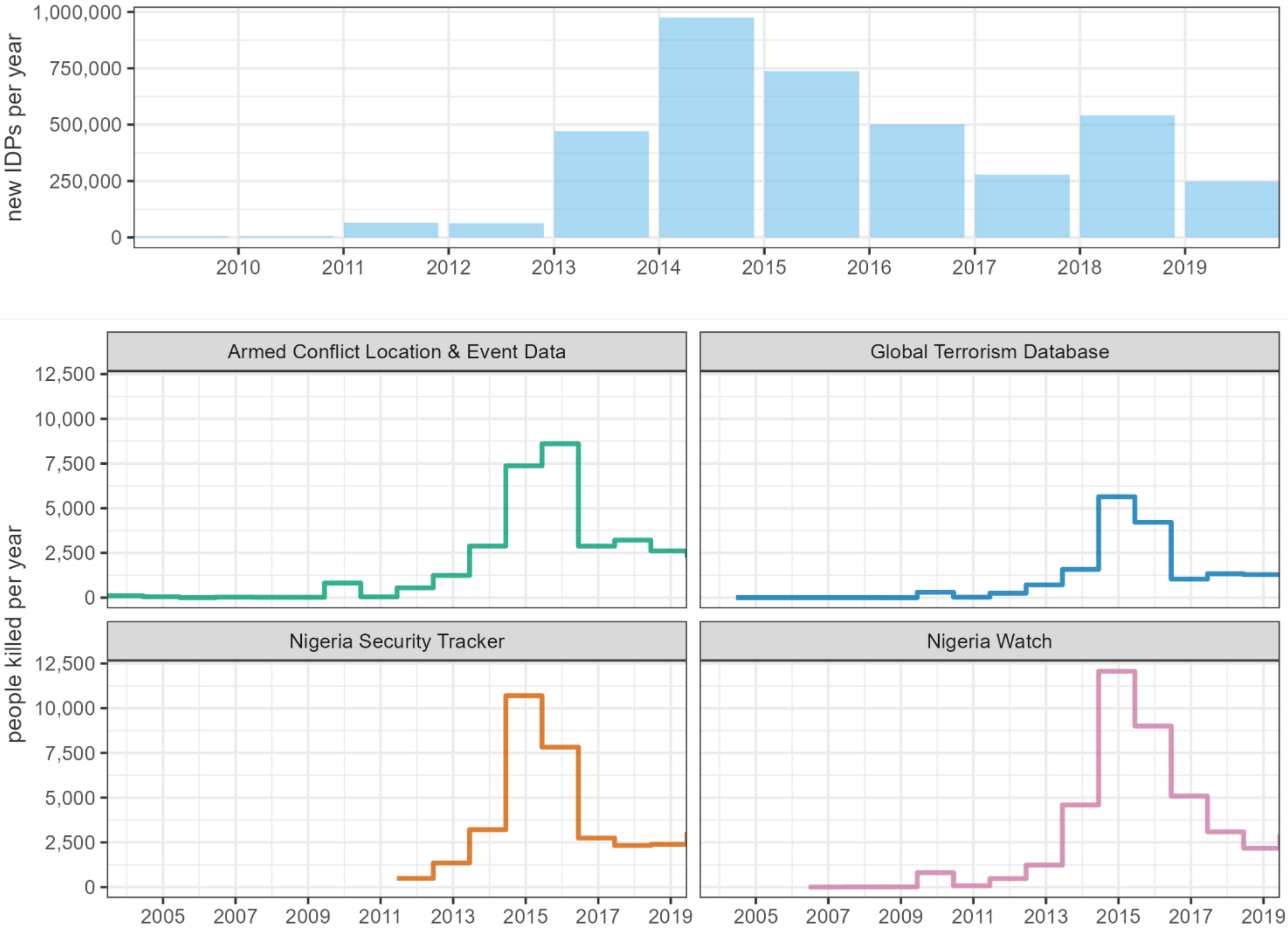

Figure S1. Trends in the number of new internally displaced persons (IDPs) due to conflict and people killed per year (by source). IDP numbers are as published by the Internal Displacement Monitoring Centre [1], and include displacement anywhere in Nigeria.

Methods

Geographic strata

Table S1 lists the different geographic units associated with each LGA, including state, domain (used as the sampling universe of most mortality surveys) and nearest market.

Table S1. List of geographic units and equivalencies.

state	domain	subdomain	LGA	market	criterion for assigning the LGA to the market
Adamawa	Southern Adamawa	Southern Adamawa	**Demsa**	Mubi	Closest market to the LGA
Adamawa	Southern Adamawa	Southern Adamawa	**Fufore**	Mubi	Closest market to the LGA
Adamawa	Southern Adamawa	Southern Adamawa	**Ganaye**	Mubi	Closest market to the LGA
Adamawa	Northern Adamawa	Northern Adamawa B	**Gireri**	Mubi	Same domain as the market
Adamawa	Southern Adamawa	Southern Adamawa	**Gombi**	Mubi	Closest market to the LGA
Adamawa	Southern Adamawa	Southern Adamawa	**Guyuk**	Mubi	Closest market to the LGA
Adamawa	Northern Adamawa	Northern Adamawa B	**Hong**	Mubi	Same domain as the market
Adamawa	Southern Adamawa	Southern Adamawa	**Jada**	Mubi	Closest market to the LGA
Adamawa	Southern Adamawa	Southern Adamawa	**Lamurde**	Mubi	Closest market to the LGA
Adamawa	Northern Adamawa	Northern Adamawa A	**Madagali**	Mubi	Same domain as the market
Adamawa	Northern Adamawa	Northern Adamawa A	**Maiha**	Mubi	Same domain as the market
Adamawa	Southern Adamawa	Southern Adamawa	**Mayo Belwa**	Mubi	Closest market to the LGA
Adamawa	Northern Adamawa	Northern Adamawa A	**Michika**	Mubi	Same domain as the market
Adamawa	Northern Adamawa	Northern Adamawa A	**Mubi North**	Mubi	Same domain as the market
Adamawa	Northern Adamawa	Northern Adamawa B	**Mubi South**	Mubi	Same domain as the market
Adamawa	Southern Adamawa	Southern Adamawa	**Numan**	Mubi	Closest market to the LGA
Adamawa	Southern Adamawa	Southern Adamawa	**Shelleng**	Mubi	Closest market to the LGA
Adamawa	Northern Adamawa	Northern Adamawa B	**Song**	Mubi	Same domain as the market
Adamawa	Southern Adamawa	Southern Adamawa	**Toungo**	Mubi	Closest market to the LGA
Adamawa	Southern Adamawa	Southern Adamawa	**Yola North**	Mubi	Closest market to the LGA
Adamawa	Southern Adamawa	Southern Adamawa	**Yola South**	Mubi	Closest market to the LGA
Borno	Northern Borno	Northern Borno	**Abadam**	Maiduguri	Closest market to the LGA
Borno	Southern Borno	Southern Borno	**Askira Uba**	Biu	Same domain as the market
Borno	Eastern Borno	Eastern Borno	**Bama**	Maiduguri	Closest market to the LGA
Borno	Southern Borno	Southern Borno	**Bayo**	Biu	Same domain as the market
Borno	Southern Borno	Southern Borno	**Biu**	Biu	Same domain as the market
Borno	Southern Borno	Southern Borno	**Chibok**	Biu	Same domain as the market
Borno	Central Borno	Central Borno A	**Damboa**	Maiduguri	Closest market to the LGA
Borno	Eastern Borno	Eastern Borno	**Dikwa**	Maiduguri	Closest market to the LGA
Borno	Central Borno	Central Borno B	**Gubio**	Maiduguri	Closest market to the LGA
Borno	Northern Borno	Northern Borno	**Guzamala**	Maiduguri	Closest market to the LGA
Borno	Eastern Borno	Eastern Borno	**Gwoza**	Maiduguri	Closest market to the LGA
Borno	Southern Borno	Southern Borno	**Hawul**	Biu	Same domain as the market
Borno	MMC/Jere	MMC/Jere	**Jere**	Maiduguri	Same domain as the market
Borno	Central Borno	Central Borno A	**Kaga**	Maiduguri	Closest market to the LGA
Borno	Eastern Borno	Eastern Borno	**Kala Balge**	Maiduguri	Closest market to the LGA
Borno	Central Borno	Central Borno A	**Konduga**	Maiduguri	Closest market to the LGA
Borno	Northern Borno	Northern Borno	**Kukawa**	Maiduguri	Closest market to the LGA
Borno	Southern Borno	Southern Borno	**Kwaya Kusar**	Biu	Same domain as the market
Borno	Central Borno	Central Borno B	**Mafa**	Maiduguri	Closest market to the LGA
Borno	Central Borno	Central Borno B	**Magumeri**	Maiduguri	Closest market to the LGA

state	domain	subdomain	LGA	market	criterion for assigning the LGA to the market
Borno	MMC/Jere	MMC/Jere	**Maiduguri**	Maiduguri	Same domain as the market
Borno	Central Borno	Central Borno B	**Marte**	Maiduguri	Closest market to the LGA
Borno	Northern Borno	Northern Borno	**Mobbar**	Maiduguri	Closest market to the LGA
Borno	Central Borno	Central Borno B	**Monguno**	Maiduguri	Closest market to the LGA
Borno	Eastern Borno	Eastern Borno	**Ngala**	Maiduguri	Closest market to the LGA
Borno	Northern Borno	Northern Borno	**Nganzai**	Maiduguri	Closest market to the LGA
Borno	Southern Borno	Southern Borno	**Shani**	Biu	Same domain as the market
Yobe	Central Yobe	Central Yobe	**Bade**	Potiskum	Closest market to the LGA
Yobe	Central Yobe	Central Yobe	**Bursari**	Damaturu	Closest market to the LGA
Yobe	Southern Yobe	Southern Yobe A	**Damaturu**	Damaturu	Closest market to the LGA
Yobe	Southern Yobe	Southern Yobe A	**Fika**	Potiskum	Closest market to the LGA
Yobe	Southern Yobe	Southern Yobe A	**Fune**	Potiskum	Closest market to the LGA
Yobe	Central Yobe	Central Yobe	**Geidam**	Damaturu	Closest market to the LGA
Yobe	Southern Yobe	Southern Yobe B	**Gujba**	Damaturu	Closest market to the LGA
Yobe	Southern Yobe	Southern Yobe B	**Gulani**	Potiskum	Closest market to the LGA
Yobe	Central Yobe	Central Yobe	**Jakusko**	Potiskum	Closest market to the LGA
Yobe	Northern Yobe	Northern Yobe A	**Karasuwa**	Potiskum	Closest market to the LGA
Yobe	Northern Yobe	Northern Yobe B	**Machina**	Potiskum	Closest market to the LGA
Yobe	Southern Yobe	Southern Yobe A	**Nangere**	Potiskum	Closest market to the LGA
Yobe	Northern Yobe	Northern Yobe A	**Nguru**	Potiskum	Closest market to the LGA
Yobe	Southern Yobe	Southern Yobe A	**Potiskum**	Potiskum	Closest market to the LGA
Yobe	Southern Yobe	Southern Yobe A	**Tarmua**	Damaturu	Closest market to the LGA
Yobe	Northern Yobe	Northern Yobe B	**Yunusari**	Potiskum	Closest market to the LGA
Yobe	Northern Yobe	Northern Yobe B	**Yusufari**	Potiskum	Closest market to the LGA

SMART mortality surveys

Table S2 shows key characteristics of each of the 70 SMART mortality surveys included in the analysis. Figure S2 shows the LGA-month coverage of the surveys. No surveys could be acquired in 2019 due to the time-limited extent of approvals to access data. We otherwise believe that, with the exception of a small number of surveys done by Médecins Sans Frontières in IDP settlements, we were able to secure the vast majority of mortality data available during 2016-2018.

We cleaned each dataset to remove obviously wrong entries and re-analysed each survey using a fixed-effects Poisson regression, with standard errors adjusted for intra-cluster correlation as per the R `survey` package. We computed a survey weight in the range 0 to 1 for each observation, equal to the anthropometric quality score automatically generated by ENA software during validation of each survey (this score considers various patterns in the data suggestive of adequate staff training and fieldwork [2]; we normalised scores to 1; see Figure S3), multiplied by the proportion of the intended sampling universe that was actually reached during the survey, as per survey report (or 1.0 if the entire sampling universe was reached).

Table S2. Detail of individual survey characteristics.

N	State	Domain	LGA	Start date	End date	N households	N individuals	Household size	Days in recall period	Quality score	Sampling coverage	Crude birth rate†	Net migration rate†	Crude death rate‡	Under 5y death rate‡
1	Borno	Central Borno	Konduga	16/07/2016	20/07/2016	412	2483	6.0	116	0.86	1.00	17.9	23.1	0.39	0.53
2	Borno	Central Borno	Kaga	25/07/2016	29/07/2016	406	2224	5.4	100	0.94	1.00	26.4	23.1	0.54	0.95
3	Borno	MCC/Jere		17/10/2016	21/10/2016	549	3225	5.6	292	0.88	0.93	34.9	-21.0	0.29	0.75
4	Adamawa	Northern Adamawa		17/10/2016	27/10/2016	540	3080	5.3	295	0.90	0.93	34.1	-52.3	0.41	0.94
5	Yobe	Southern Yobe		17/10/2016	11/11/2016	555	3391	5.8	302	0.70	0.90	38.1	-27.4	0.33	0.65
6	Adamawa	Southern Adamawa		19/10/2016	01/11/2016	566	3109	5.1	298	0.66	0.97	37.4	-25.6	0.40	0.97
7	Borno	Southern Borno		23/10/2016	28/10/2016	592	3506	5.7	298	0.64	1.00	40.7	-23.1	0.25	0.94
8	Borno	Central Borno		28/10/2016	03/11/2016	582	2995	4.9	304	0.82	0.88	42.1	2.4	0.52	1.57
9	Yobe	Northern Yobe		03/11/2016	11/11/2016	319	1637	4.9	309	0.58	0.97	44.4	-8.1	0.48	1.77
10	Yobe	Central Yobe		05/11/2016	10/11/2016	557	3259	5.5	303	0.86	0.93	44.6	-30.9	0.60	1.89
11	Borno	Central Borno	Monguno	23/11/2016	27/11/2016	412	2317	5.2	90	0.80	0.50	40.9	133.3	1.36	1.90
12	Borno	Eastern Borno	Bama	09/12/2016	13/12/2016	395	1545	3.8	90	0.90	0.50	18.3	26.2	1.58	0.73
13	Borno	Eastern Borno	Dikwa	10/12/2016	14/12/2016	395	1987	4.9	90	0.88	0.50	30.8	59.5	1.24	0.78
14	Yobe	Southern Yobe		13/02/2017	08/03/2017	619	3753	5.8	164	0.88	0.94	44.9	-32.5	0.89	2.18
15	Yobe	Southern Yobe		14/02/2017	08/03/2017	578	3008	4.9	165	0.96	1.00	61.8	-78.6	0.84	1.62
16	Yobe	Central Yobe		18/02/2017	05/03/2017	539	3214	5.8	160	0.86	1.00	22.0	-50.5	1.12	2.44
17	Borno	MCC/Jere		20/02/2017	24/02/2017	594	3618	5.8	162	0.96	0.97	40.0	-67.6	0.81	1.38
18	Yobe	Northern Yobe		22/02/2017	04/03/2017	579	3090	5.1	167	0.86	0.94	21.7	-25.9	0.98	2.52
19	Borno	MCC/Jere	Jere	23/02/2017	10/03/2017	617	3639	5.4	170	0.98	0.08	35.5	-109.9	1.46	3.01
20	Borno	Central Borno		24/02/2017	29/03/2017	537	2753	4.8	168	0.80	0.87	55.2	-73.8	1.28	2.43
21	Borno	Southern Borno		25/02/2017	02/03/2017	600	3567	5.6	162	0.76	0.97	45.9	-103.1	0.53	1.87
22	Borno	Central Borno	Konduga	10/03/2017	12/03/2017	539	2959	5.0	179	1.00	0.08	38.2	-119.8	0.95	1.75
23	Borno	Northern Borno		14/03/2017	29/03/2017	400	1923	4.5	190	0.92	0.65	37.2	-70.4	1.26	2.05
24	Borno	Eastern Borno	Ngala	21/03/2017	23/03/2017	600	2974	4.6	190	0.90	0.39	33.4	-75.0	1.45	3.73
25	Yobe	Southern Yobe		04/07/2017	13/07/2017	670	3880	5.5	99	0.70	0.94	76.3	-83.8	1.57	4.46
26	Yobe	Central Yobe		05/07/2017	13/07/2017	665	3651	5.3	100	0.74	0.97	40.7	-68.4	0.84	1.96
27	Yobe	Northern Yobe		08/07/2017	12/07/2017	680	3664	5.2	101	0.84	0.94	73.8	-46.2	1.27	2.99
28	Borno	Central Borno		16/07/2017	07/08/2017	779	3904	4.9	118	0.86	0.89	41.7	-37.8	0.86	1.73
29	Borno	Southern Borno		16/07/2017	25/07/2017	852	4997	5.7	111	0.78	0.97	30.8	-38.0	0.38	1.10
30	Borno	Northern Borno		24/07/2017	06/08/2017	553	2488	4.3	121	0.70	0.70	40.5	-63.2	0.95	2.29
31	Borno	MCC/Jere		27/07/2017	31/07/2017	839	4936	5.7	120	0.76	0.97	22.7	-39.2	0.42	0.96
32	Borno	Eastern Borno		02/08/2017	07/08/2017	803	4353	5.3	125	0.86	0.92	29.1	-94.8	0.36	0.95
33	Yobe	Southern Yobe	Gulani	17/10/2017	23/10/2017	504	3166	6.2	116	0.86	0.92	17.9	0.0	0.79	1.54
34	Yobe	Northern Yobe	Yunusari	26/10/2017	31/10/2017	441	2659	5.7	123	1.00	0.64	14.2	-112.7	0.54	1.74
35	Yobe	Central Yobe		06/11/2017	14/11/2017	544	3037	5.4	103	0.78	0.91	82.7	-68.7	0.83	1.98
36	Yobe	Southern Yobe		06/11/2017	14/11/2017	631	3721	5.8	103	0.78	0.97	43.8	-35.2	0.42	1.14
37	Yobe	Northern Yobe		07/11/2017	13/11/2017	615	2955	4.6	102	0.86	0.97	74.3	-99.5	1.28	3.43
38	Adamawa	Northern Adamawa		17/11/2017	23/11/2017	643	3370	5.2	113	0.96	0.97	56.9	0.0	0.55	1.32
39	Adamawa	Southern Adamawa		17/11/2017	23/11/2017	607	3029	4.8	113	0.72	0.97	63.8	-70.1	0.47	0.94
40	Borno	Southern Borno		24/11/2017	28/11/2017	643	3712	5.6	119	0.86	1.00	52.9	-30.6	0.59	1.32
41	Borno	Central Borno		27/11/2017	07/12/2017	615	3328	5.3	125	0.96	0.94	60.7	-30.8	0.51	1.20
42	Borno	MCC/Jere		30/11/2017	03/12/2017	633	4069	6.3	137	0.96	0.94	32.1	-20.3	0.29	0.56
43	Borno	Northern Borno		04/12/2017	18/12/2017	592	2784	4.5	133	0.92	0.97	49.0	-55.8	0.78	2.40
44	Borno	Eastern Borno		07/12/2017	21/12/2017	639	3388	5.2	137	0.96	0.94	63.6	-22.8	0.34	0.75
45	Yobe	Southern Yobe		16/04/2018	26/04/2018	575	3741	6.3	118	0.96	0.95	23.8	-50.9	0.43	0.80
46	Yobe	Central Yobe		17/04/2018	23/04/2018	678	3893	5.6	120	0.78	0.92	20.9	-55.8	0.51	1.48
47	Adamawa	Northern Adamawa		17/04/2018	07/05/2018	149	826	7.3	116	0.96	0.97	26.3	-62.3	0.55	0.85
48	Yobe	Northern Yobe		19/04/2018	07/05/2018	670	3480	5.0	119	0.80	0.97	25.6	-51.2	0.49	0.78

N	State	Domain	LGA	Start date	End date	N households	N individuals	Household size	Days in recall period	Quality score	Sampling coverage	Crude birth rate†	Net migration rate†	Crude death rate‡	Under 5y death rate‡
49	Adamawa	Southern Adamawa		29/04/2018	04/05/2018	715	3537	4.8	128	0.92	0.97	10.4	-37.7	0.18	0.40
50	Yobe	Northern Yobe	Machina	07/05/2018	11/05/2018	677	3274	4.7	93	0.80	0.96	79.3	-51.6	0.36	0.60
51	Borno	Southern Borno		07/05/2018	27/05/2018	631	3452	5.4	116	0.94	1.00	18.5	-16.8	0.31	0.52
52	Borno	Central Borno		12/05/2018	29/05/2018	441	2330	5.2	116	0.70	0.96	13.4	-33.5	0.40	0.82
53	Borno	MCC/Jere		12/05/2018	20/05/2018	461	2790	6.0	143	0.96	1.00	7.3	-20.0	0.25	0.43
54	Yobe	Northern Yobe	Yunusari	14/05/2018	19/05/2018	698	3379	4.8	93	0.86	0.96	79.1	-51.2	0.32	0.72
55	Borno	Northern Borno		15/05/2018	24/05/2018	350	1612	4.5	146	0.84	0.61	7.7	-18.5	0.17	0.23
56	Yobe	Southern Yobe	Fika	21/05/2018	26/05/2018	716	4064	5.6	93	1.00	0.95	90.3	-36.9	0.19	0.52
57	Borno	Eastern Borno		22/05/2018	25/05/2018	456	2428	5.1	150	0.92	0.82	16.8	-77.1	0.22	0.64
58	Borno	Central Borno	Monguno	28/05/2018	30/05/2018	490	2232	4.5	156	0.92	0.91	15.7	-5.2	0.17	0.29
59	Borno	Northern Borno		15/09/2018	20/09/2018	397	1809	4.4	155	0.64	0.64	24.4	-54.0	0.67	0.61
60	Yobe	Southern Yobe		19/09/2018	10/10/2018	489	3008	5.9	142	0.82	0.97	23.5	-89.9	0.55	1.02
61	Adamawa	Northern Adamawa		21/09/2018	30/09/2018	466	2561	5.3	132	0.90	0.90	27.9	-33.3	0.62	1.14
62	Adamawa	Southern Adamawa		21/09/2018	26/09/2018	628	3383	5.1	130	0.64	1.00	18.9	-37.8	1.06	1.91
63	Yobe	Central Yobe		23/09/2018	08/10/2018	517	3218	6.0	145	0.82	0.91	26.2	-81.1	0.32	0.49
64	Adamawa	Northern Adamawa		28/09/2018	01/10/2018	510	3028	5.8	136	0.96	0.90	8.8	-46.5	0.53	1.53
65	Yobe	Northern Yobe		28/09/2018	05/10/2018	647	3844	5.7	142	0.78	1.00	28.3	-84.3	0.65	0.80
66	Yobe	Northern Yobe		28/09/2018	10/10/2018	592	3216	5.2	142	0.70	0.94	38.9	-42.1	0.89	2.25
67	Borno	Southern Borno		01/10/2018	06/10/2018	584	3468	5.8	140	0.92	0.97	24.8	-19.5	0.31	0.50
68	Borno	Central Borno		04/10/2018	15/10/2018	635	3088	4.7	146	0.86	0.84	25.6	-53.6	0.88	1.75
69	Borno	MCC/Jere		08/10/2018	14/10/2018	578	3882	6.5	148	0.86	1.00	18.8	-53.4	0.65	1.33
70	Borno	Central Borno		11/10/2018	22/10/2018	661	3306	4.8	153	0.98	1.00	23.0	-41.8	0.57	1.00

† per 1000 person-years. ‡ per 10,000 person/child-days.

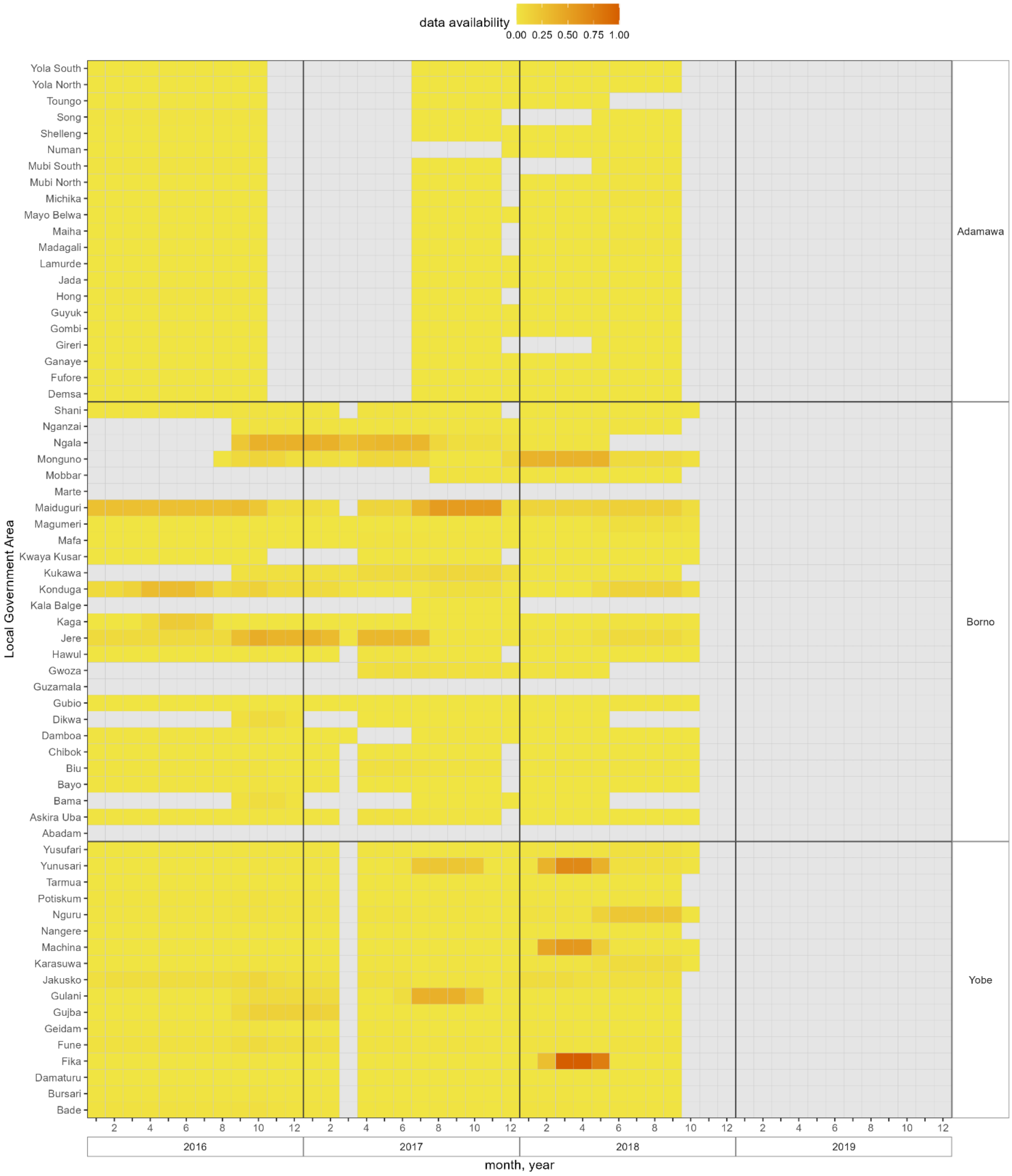

Figure S2. Coverage of SMART mortality surveys, by state and LGA. Heat colours denote months falling within the recall period of one or more surveys, with increasing colour intensity proportional to a data availability index corresponding to (proportion of month covered) x (survey person-time falling within the LGA-month) x (survey quality score) x (survey sampling coverage), rescaled to unity to provide a relative scale for the amount of information available for each survey-month. Grey LGA-months are those not covered by any survey.

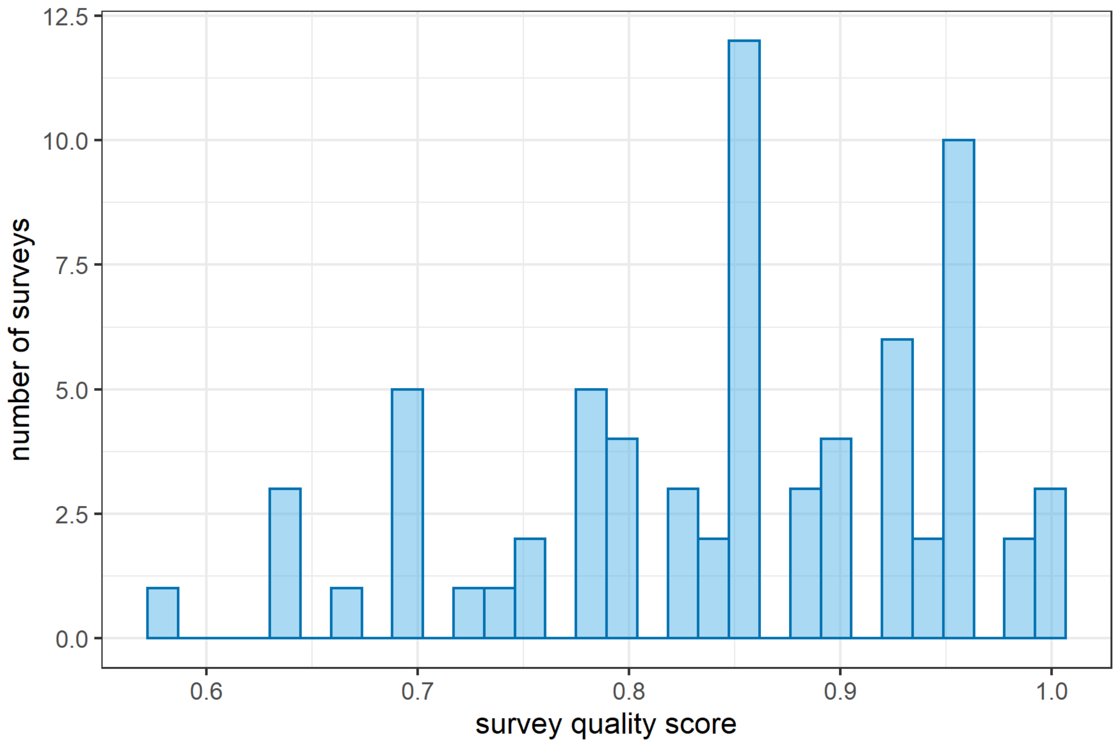

Figure S3. Distribution of quality score of SMART mortality surveys included in the analysis.

Reconstruction of population denominators

Table S3 lists the different sources of information we drew on to estimate the number of people present in any LGA during any given month, as well as the number of IDPs and refugees leaving, arriving (or returning) into and currently displaced within each LGA. We defined IDPs as people who leave their home due to crisis conditions and move to somewhere else within the same LGA, to another LGA within Adamawa, Borno and Yobe or even outside of the three states, but not crossing an international border. By contrast, refugees do cross an international border.

Table S3. Sources of data on population and displacement.

Source (URL)	Reference date	Details
Population estimates		
National Bureau of Statistics (https://data.humdata.org/dataset/nigeria-2016-population-data)	June 2016	Projections of the 2006 national census, supported by humanitarian actors.
Geo-Referenced Infrastructure and Demographic Data for Development (GRID[3]) (https://grid3.gov.ng/)	August 2019	The method combines census data, ad hoc micro-census surveys, ground settlement data and remote sensing information into a statistical model, partly informed by the AfriPop approach, that generates population estimates for 100m^2 grids. It builds on previous estimation work in northern Nigeria [3].
Facebook Data for Good (https://data.humdata.org/dataset/highresolutionpopulationdensitymaps-nga)	November 2016	Population estimates for 30m2 grids, based on remote sensing approaches combined with available census and demographic survey estimates. The method is based on neural networks and rests on enumerating inhabited structures. It is explicitly conceived for use by humanitarian actors [4].

Source (URL)	Reference date	Details
Emergency Operations Centre, northeast Nigeria	November 2018	Using polio vaccination teams as the backbone, these estimates are derived by tracking settlement habitation through a combination of remote sensing and field data [5].
IDP movements		
International Organisation for Migration Displacement Tracking Matrix (https://dtm.iom.int/)	February 2015 to October 2019	Regular (mostly every two months) assessments of all known and accessible IDP settlements, collecting data on reported numbers of IDPs, their year of arrival and main LGA of origin.
Refugee movements		
United Nations High Commissioner for Refugees	January 2015 to December 2019	Approximated by the authors based on document review and triangulation of different datasets, after a systematic review of UNHCR Nigeria crisis documents on the www.reliefweb.org humanitarian document repository.

Briefly, for each LGA we forward- or back-calculated population from the time point at which each of the alternative population estimate sources was centred, using the following equation:

$$N_{t+1} = N_t(1+g) + I_{A,t} - I_{D,t} + R_{A,t} - R_{D,t}$$

where N is total population, g the assumed growth rate, I internally displaced persons and R refugees (A = arriving from another LGA, D = departing to another LGA). We then took a weighted average of the four time series arising from each population source, with the weight corresponding to a quality score comprised of various criteria [6]. Lastly, we computed the population of children under 5y using the mean proportion in this age group (20%) reported by the SMART surveys we analysed.

In order to estimate I flows, we first aggregated IDP groups within each LGA by their year of arrival and LGA of origin, as per the DTM database. Setting 1 Jan 2015 as the start date for population reconstruction, we assumed that everyone who had arrived before then was present at the start date; similarly, further arrivals during 2015 were present as of Jan 2016; and so on. We then interpolated prevalent IDP data as reported by each successive round of assessment, so as to construct monthly time series of IDPs by LGA of arrival and LGA of origin, which we used to compute flows from each LGA to other LGAs for each month. We then used these flow time series in the above equation.

As a final adjustment, we applied a correction to IDP time series whenever reconstructed population for any LGA and any alternative population source became negative, presumably because of systematic error in IDP counting (this occurred in 12/65 LGAs). The correction downward-scaled the time series so as to achieve a minimum of N = 0 for all population sources and LGAs. This was done by constraining the ratio of number of IDPs in the LGA to LGA population to ≤ 1.

Figure S4 shows trends in the estimated population by LGA, showing that the least stable patterns occurred in Borno state, where large waves of displacement and return occurred during the period. Figure S5 shows, at state level, the differences in estimates derived from each the four population sources: notably, these differences were not consistent across the three states. Lastly, Figure S6 shows the percentage of each state's population that consisted of IDPs, indicating a relatively stable pattern over the period. We separately estimate that there were 138,000 refugees from Adamawa, Borno and Yobe in neighbouring countries (Niger, Chad, Cameroon) as of January 2015. Refugee numbers had risen progressively to 239,000 by December 2019.

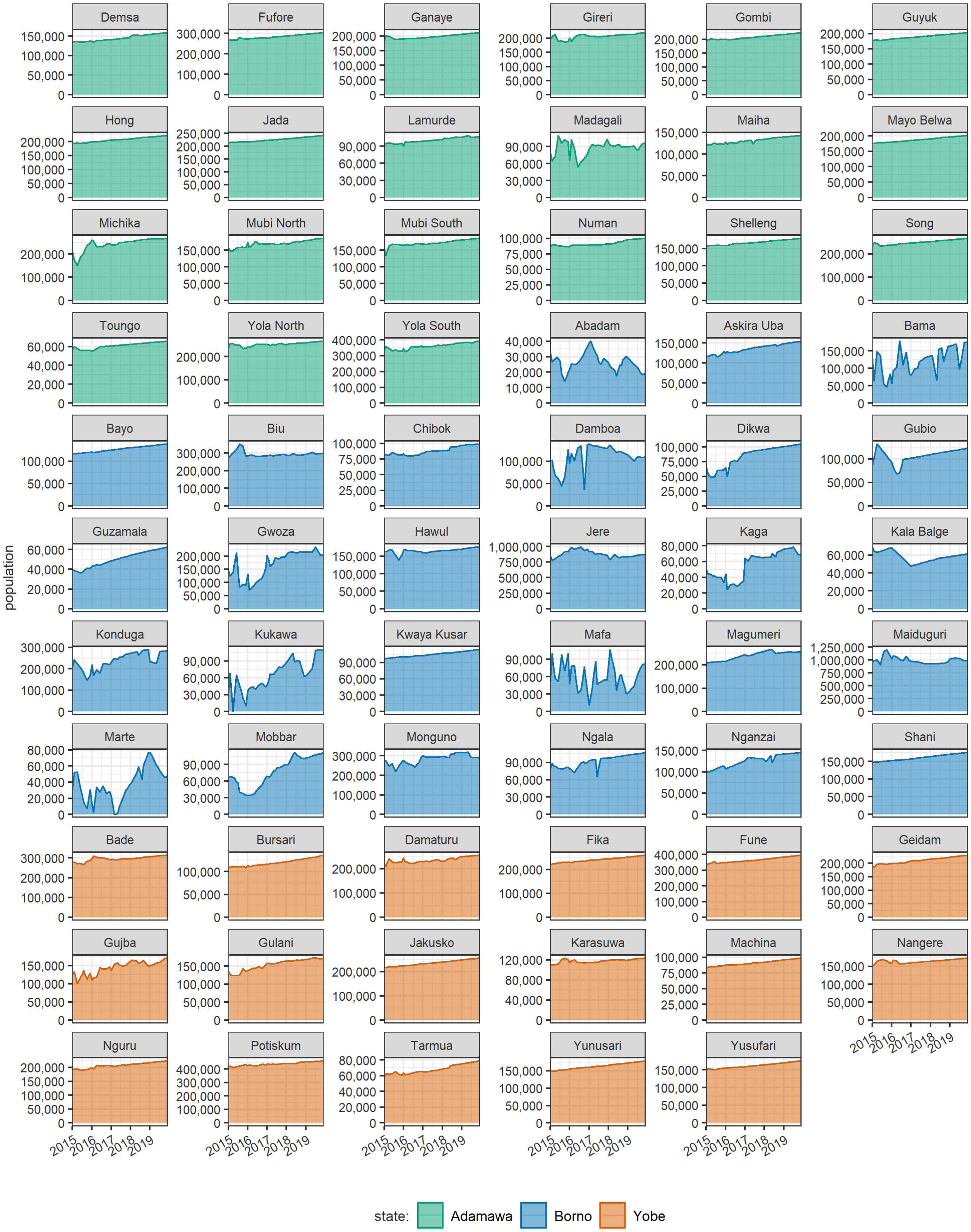

Figure S4. Evolution of estimated population, by LGA.

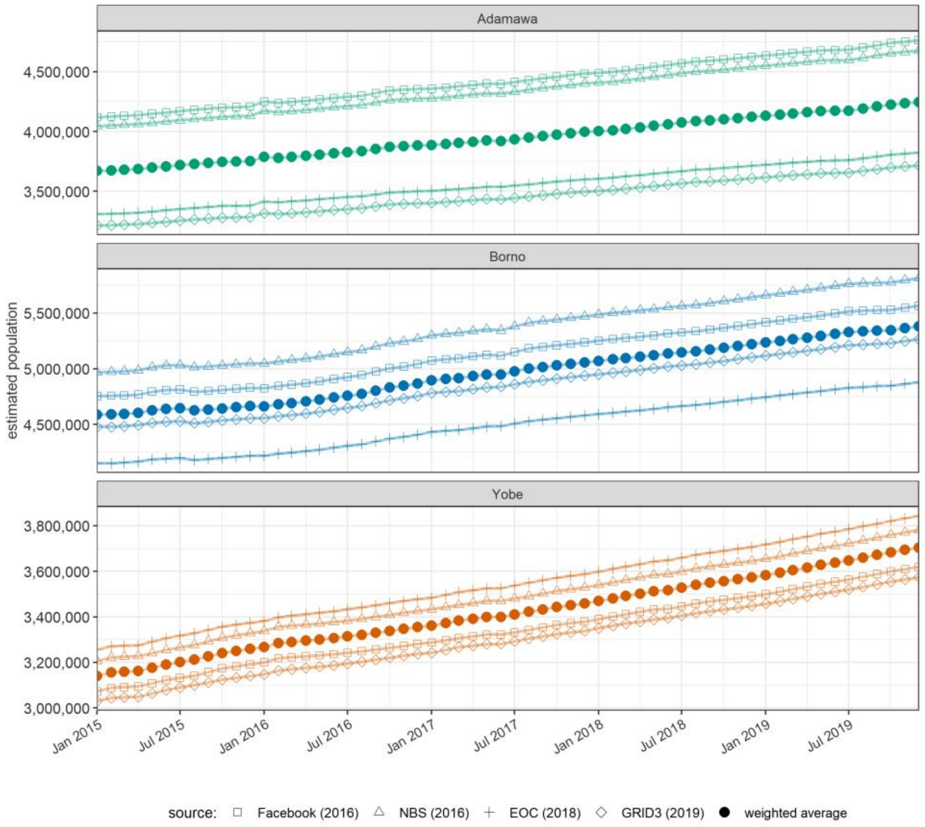

Figure S5. Evolution of estimated population, by alternative population source and state.

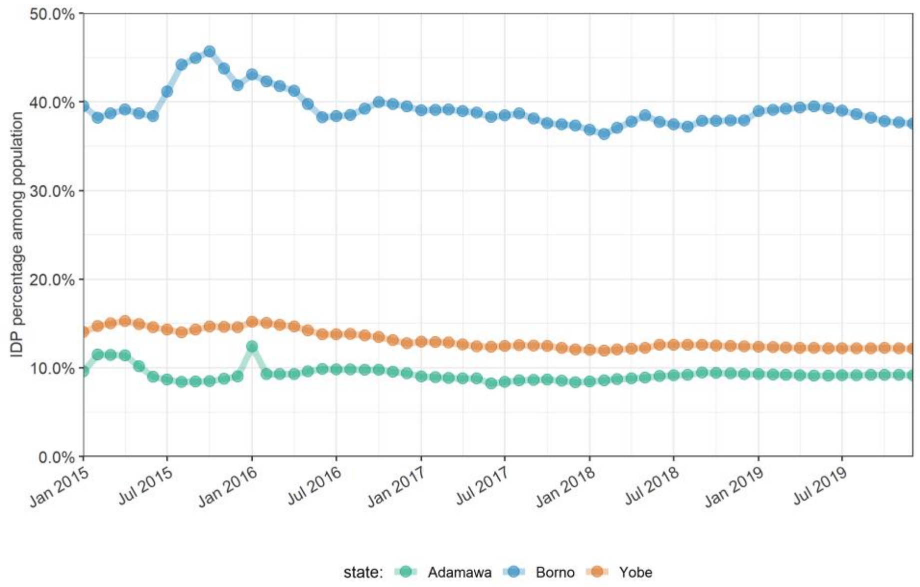

Figure S6. Evolution of the estimated proportion of the population that are IDPs, by state.

Completeness of predictor datasets

Figure S7 and Figure S8 show the completeness of candidate predictor datasets, by month (i.e. proportion of LGAs that have complete data for any given month) and LGA (i.e. proportion of months for which a given LGA has a complete data), respectively.

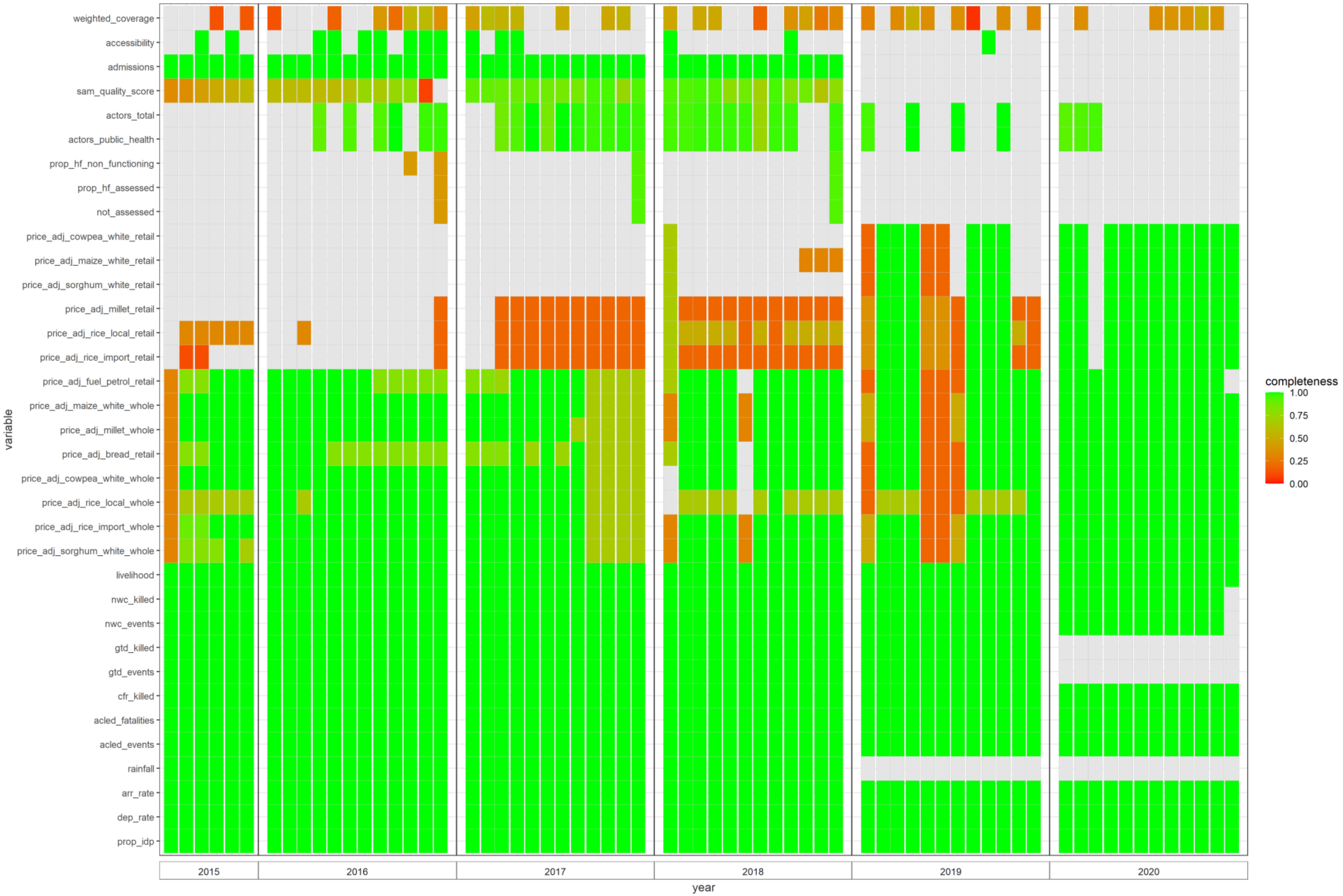

Figure S7. Completeness of candidate predictor datasets, by month, prior to imputation, interpolation or smoothing.

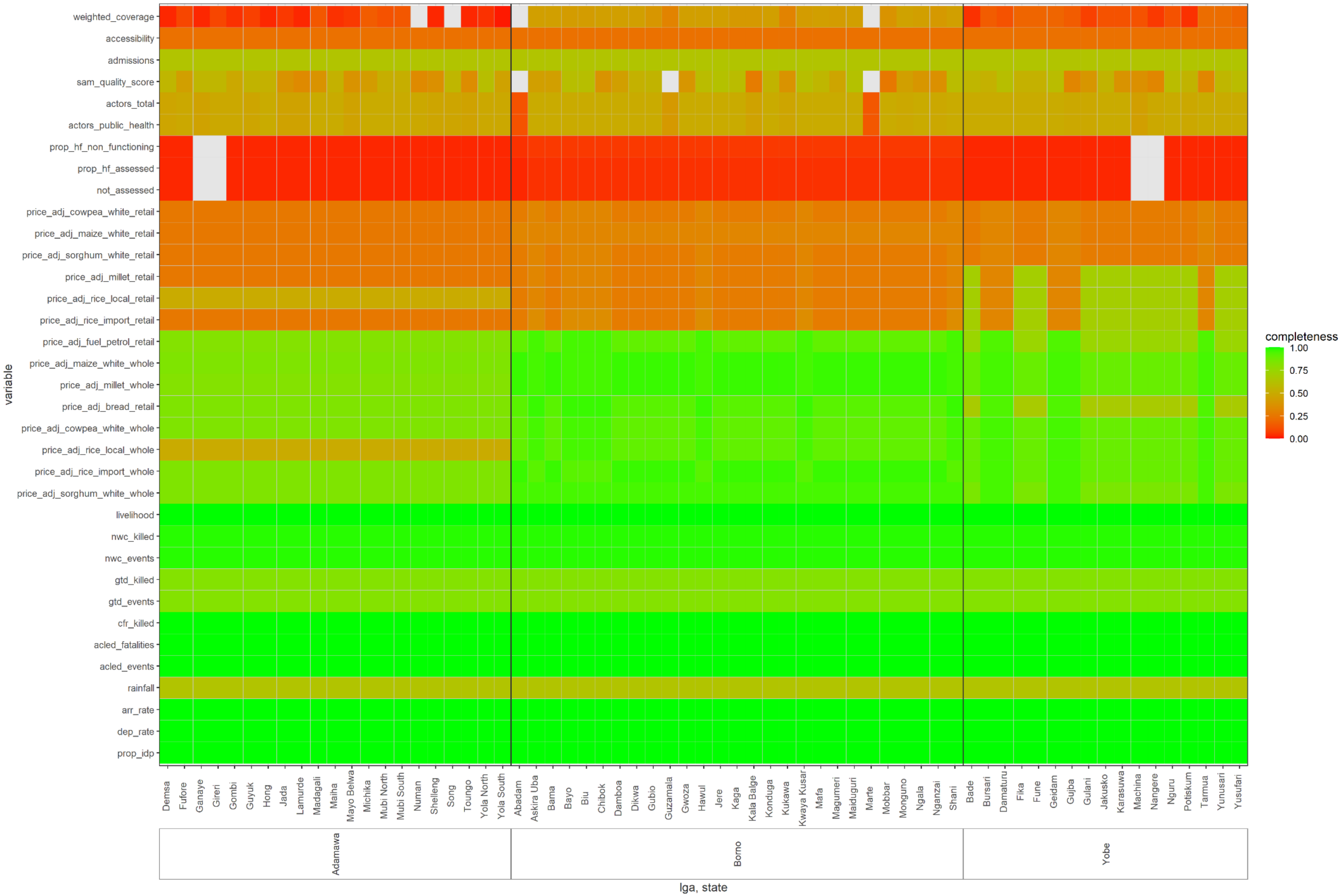

Figure S8. Completeness of candidate predictor datasets, by LGA, prior to imputation, interpolation or smoothing.

Management of predictor datasets

Market prices

Data management. The World Food Programme's dataset of market prices consists of monthly observations from a purposive selection of markets across Nigeria, with relatively more markets being followed in crisis-affected states. While data are available from 2002, in Adamawa, Borno and Yobe states they are very sparse prior to 2015.

We selected the most frequently reported-on commodities in the dataset: gasoline fuel, bread, white cowpea, white maize, millet, local rice, imported rice and white sorghum. For some of these, both wholesale and retail prices were available.

We computed an inflation-adjusted standardised price for 1Kg of each cereal staple, 1L of fuel and 1 loaf of bread as follows. First, we divided reported cereal prices by their unit to come up with a standardised price per Kg. Second, we converted the price to USD equivalent using historical monthly NGN to USD exchange rates obtained from https://fxtop.com/en/historical-exchange-rates.php . Lastly, we adjusted the price for USD inflation (https://www.in2013dollars.com/us/inflation/2010?amount=1) to come up with a price in 2010 USD.

While 12 individual markets were featured in the database for Adamawa, Borno and Yobe states, in practice only five had continuous or at least intermittent data coverage from 2015 onwards: Mubi (Adamawa), Biu and Maiduguri (Borno), Damaturu and Potiskum (Yobe). We assigned each LGA to one of these markets based on proximity criteria (Table S1). Each LGA price series was subjected to moderate spline smoothing to correct for possible data entry errors and interpolate between missing observations.

Setting counterfactual values. After inspecting the market-specific time series of maize price (Figure S9), we decided for our most likely scenario to take the median values for each market (and thus the LGA attributed to the market) during the low-price periods of January to June 2015 and 2018-2019: this reflects an implicit decision that the price increase should be considered part of the crisis conditions affecting north-east Nigeria. We took the minimum of each market time series as the best-case scenario, and the median of the entire series between 2015-2019 (which therefore includes the price increase period) as the worst-case scenario.

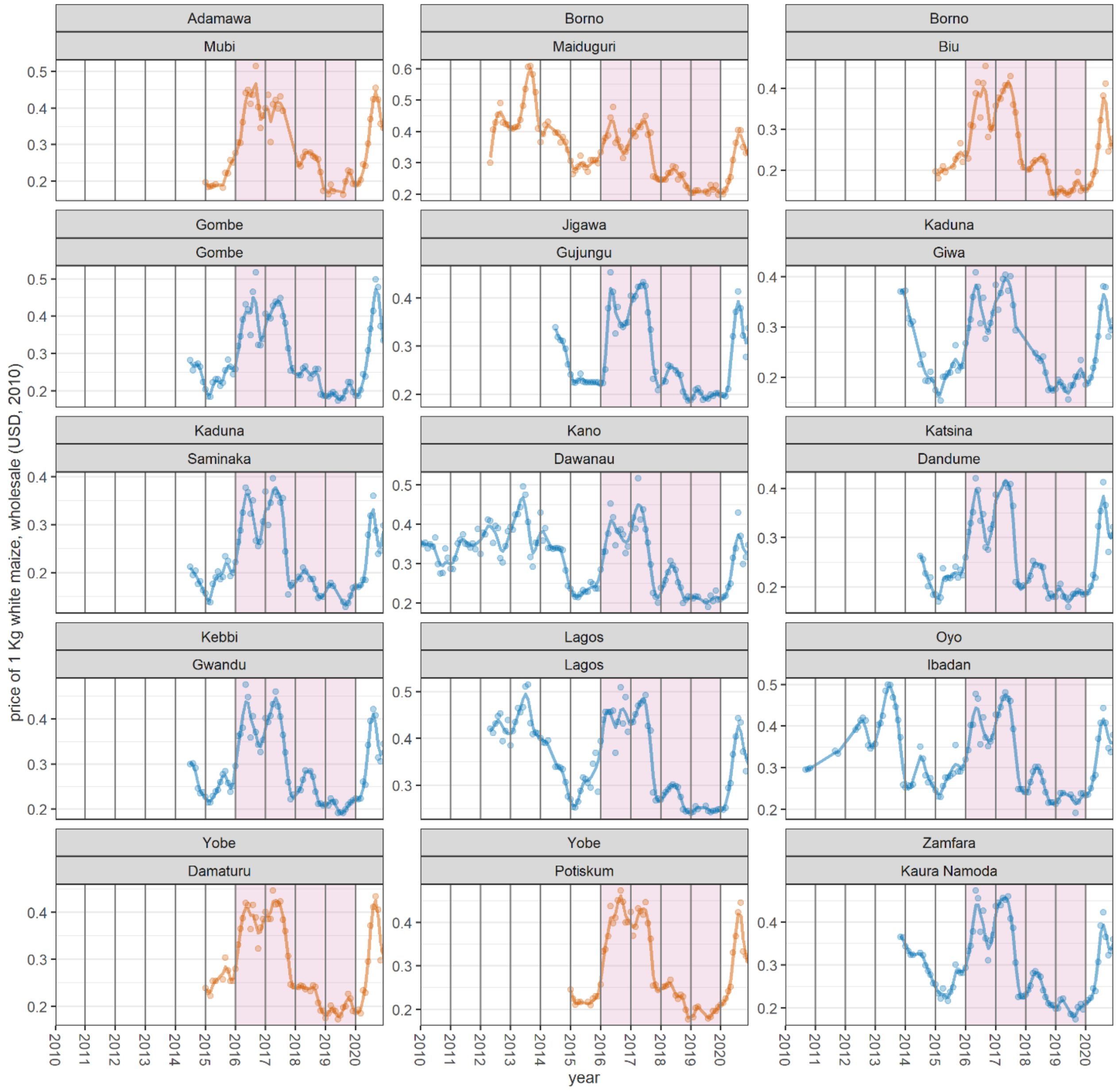

Figure S9. Trends in the price of 1Kg of white maize, at wholesale price (sale unit: 100 Kg), expressed in USD as of 2010, by market. Dots are actual observations and lines are smoothed series. Shading denotes the mortality analysis period.

Vaccination coverage

Data management. Nigeria's Vaccination Tracking System (VTS) was developed to help national and state emergency polio operations centres to estimate vaccination coverage and identify areas in need of supplemental vaccination activities. The VTS equips vaccination outreach teams with GPS devices that track the teams' movements. During their field visits, these teams also collect geographic and demographic information on settlements they visit, which is used not only to estimate vaccination coverage, but also to update population estimates. 'Vaccination geo-coverage' during any given time window is quantified as the proportion of 100 m^2 grids in targeted areas that are visited, based on GPS tracks. The system has

more recently been used during seasonal malaria chemoprophylaxis campaigns, and as such more generically produced estimates of the geo-coverage of community-based public health interventions.

We downloaded the 'time-trend-analysis' dataset from the now-defunct VTS website (http://vts.eocng.org/ChronicalCoverage/Index). The dataset is organised by ward (sub-unit within LGA and campaign (e.g. August 2017 polio mass vaccination): for each such instance, the dataset reports geo-coverage (estimated as above) and the number of 'geo-coverage denominators', roughly equivalent to similar-size population units, and which we used as weights to come up with a weighted geo-coverage by LGA and month. We then did automatic imputation to infer missing values of geo-coverage, which occurred mainly over the intervals between successive campaigns: for this we used the Random Forest method as implemented by the R `mice` package [7] (https://amices.org/mice/), with 5 chains of 20 iterations, and using all available predictors to inform the imputation.

Setting counterfactual values. To set counterfactual values, we considered geo-coverage values in states other than Adamawa, Borno and Yobe. After coming up with LGA-month weighted geo-coverage, as above, we retained LGA time series from 2012-2020 that had at least four observations. The resulting dataset contained eligible time series from 49 LGAs in Adamawa, Borno and Yobe and 161 LGAs in 11 other states (Bauchi, Gombe, Jigawa, Kaduna, Kano, Katsina, Kebbi, Kogi, Kwara, Sokoto, Zamfara). We then fitted, for both (i) Adamawa, Borno and Yobe and (ii) all the other states, a generalised additive growth model of geo-coverage (assumed to be normally distributed) as a function of increasing time (month), with time nested within LGA as a random effect, and unity-normalised geo-coverage denominator weights. Model-predicted geo-coverage is shown in Figure S10.

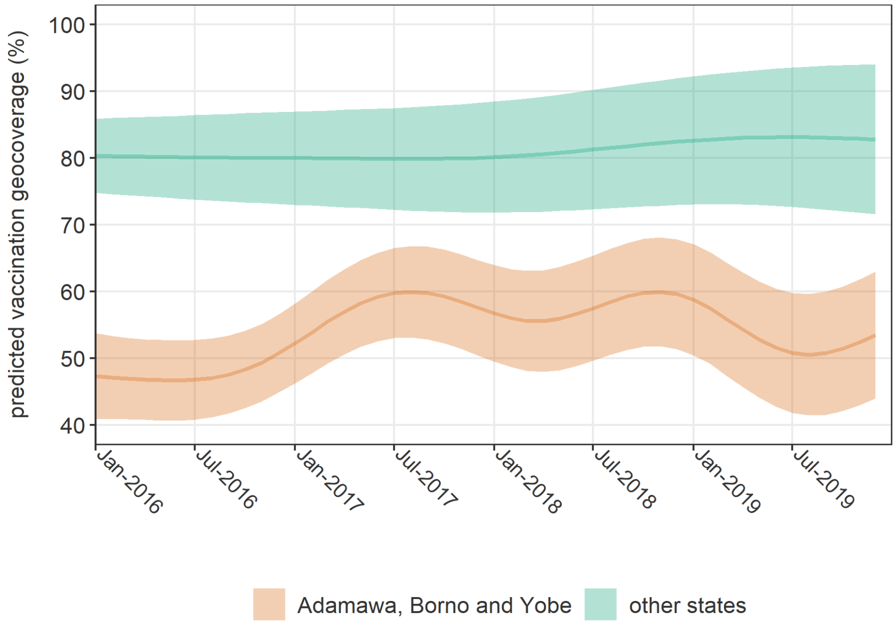

Figure S10. Estimated vaccination geo-coverage as predicted using generalised additive modelling from VTS data, for Adamawa, Borno and Yobe and other states, respectively. Shaded areas represent 95% confidence intervals.

Results

Predictive model

Figure S11 and Figure S12 show the model's out-of-sample predictive accuracy on cross-validation. In practice, both graphs represent a worst-case scenario in which prediction always takes place in LGAs that the model has not been trained on (i.e. new levels of the random effect). Because survey coverage in Nigeria is very high, in reality both models predict deaths mostly for 'within-sample' LGAs.

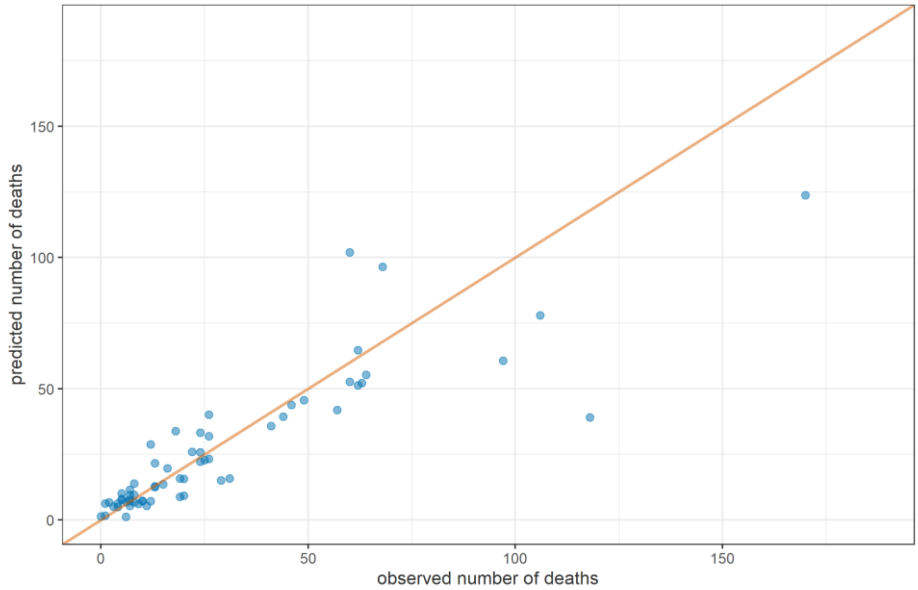

Figure S11. Predictive accuracy of the model for CDR on ten-fold cross-validation. Each blue dot shows the number of observed and predicted deaths by LGA. The red line shows perfect prediction.

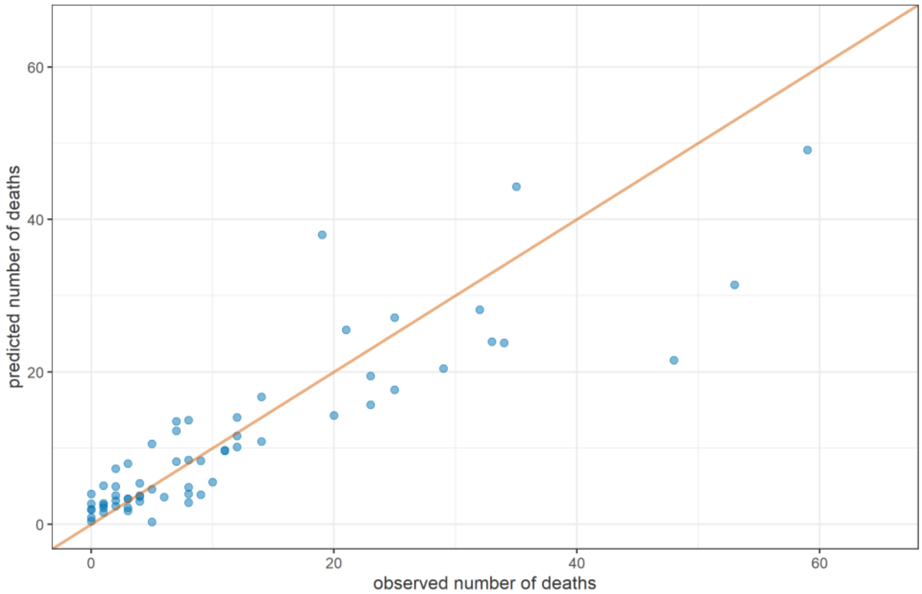

Figure S12. Predictive accuracy of the model for U5DR on ten-fold cross-validation. Each blue dot shows the number of observed and predicted deaths by LGA. The red line shows perfect prediction.

Discussion

Sensitivity analysis: bias in displacement and population data

Figure S13 shows how the central estimate (likely counterfactual scenario) of total and excess death toll varies as a function of potential bias in both the four base population estimates and the number of IDPs as reported in the DTM database.

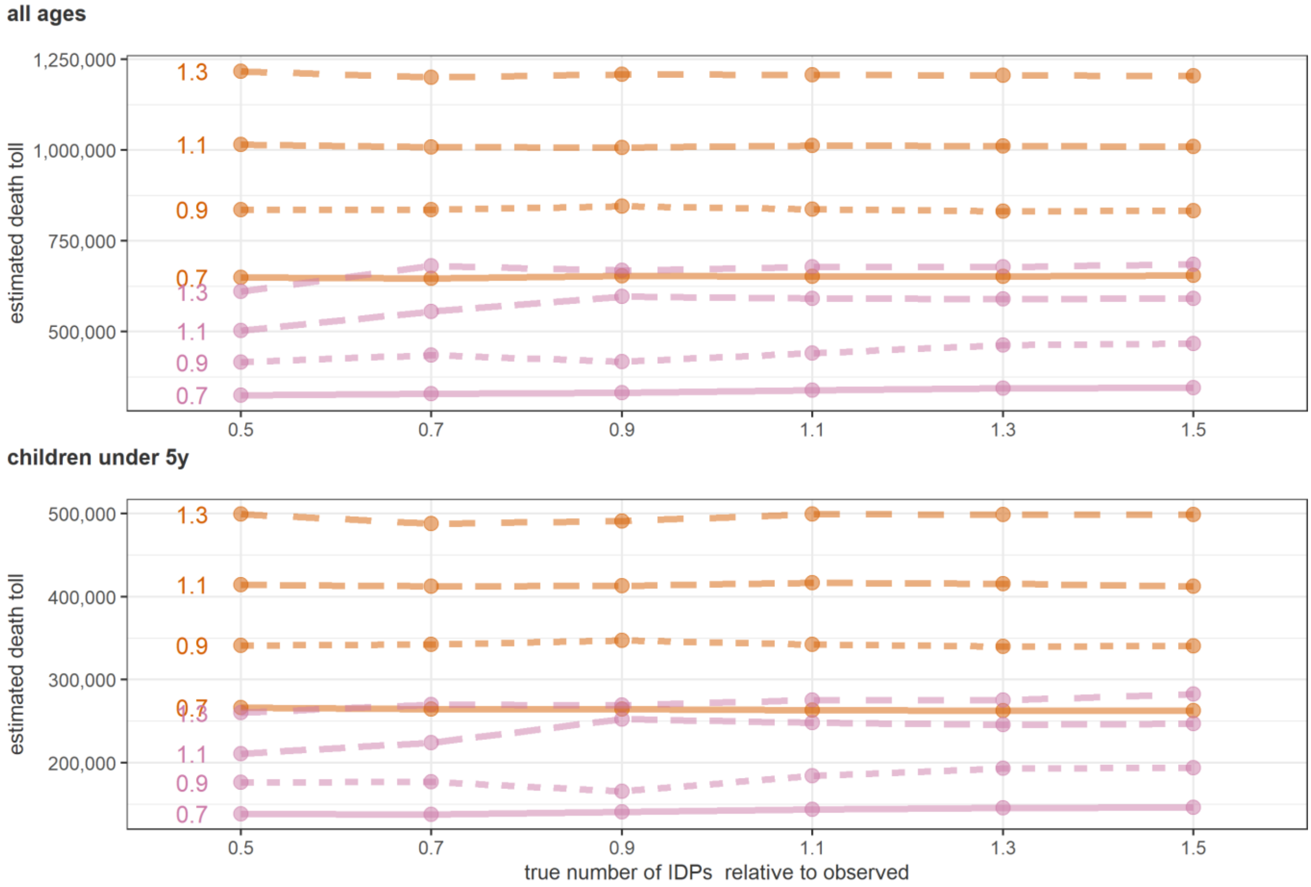

Figure S13. Sensitivity of the estimated total (orange) and excess (pink) death toll (as the point estimate given the most likely counterfactual scenario), for both all ages and children under 5y, to varying levels of bias in input population and IDP data. Each sensitivity value is a ratio of actual to observed values, i.e. multiplier applied to the observed data (accordingly, values < 1 imply under-estimation, and vice versa). Sensitivity values for the ratio of true to observed population figures are indicated within the plots to the left of the corresponding output estimates.

Sensitivity analysis: Under-estimation in U5DR

Figure S14 shows how the central estimates of total and excess death tolls, by age group, vary with increasing percentages of under 5y deaths that were not detected during SMART surveys.

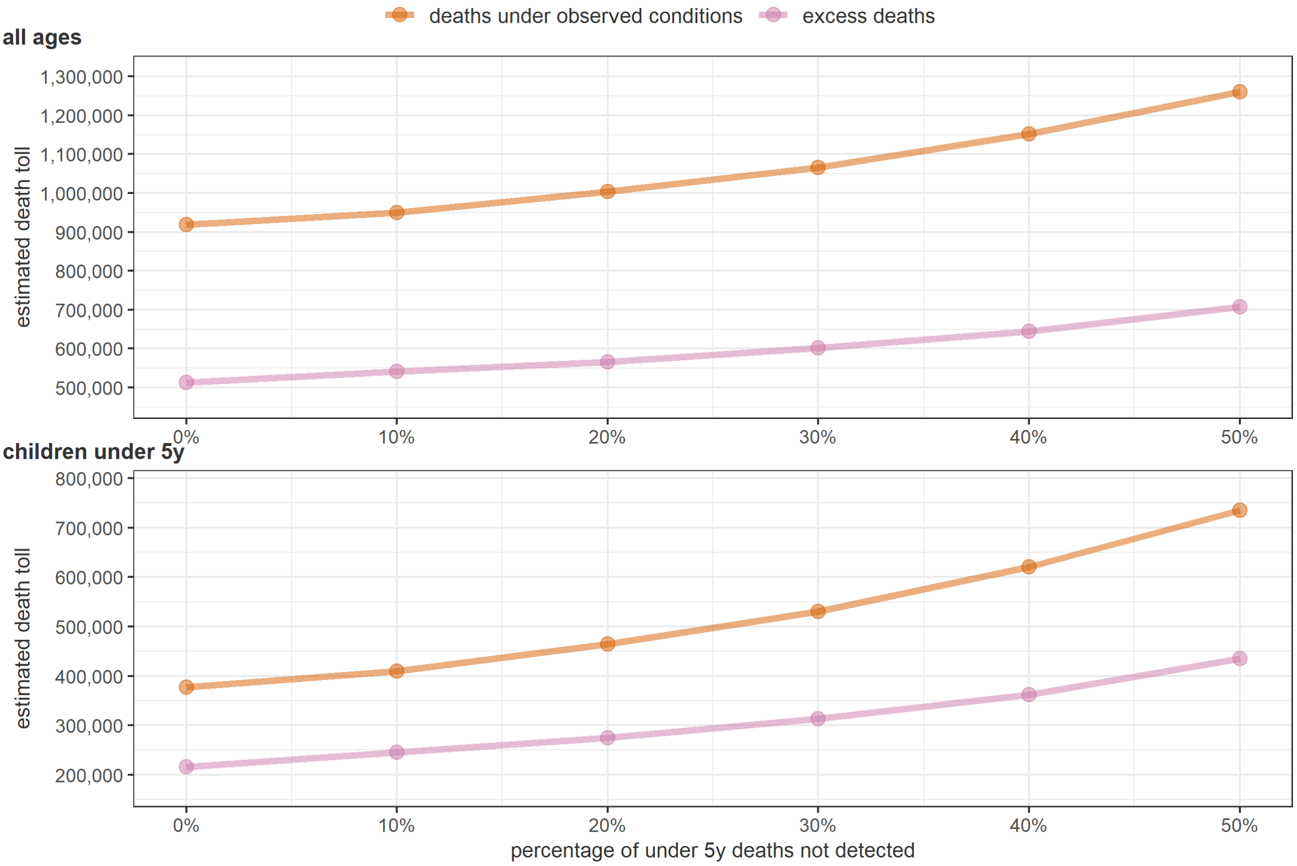

Figure S14. Sensitivity of the estimated total and excess death toll (as point estimates given the most likely counterfactual scenario), for both all ages and children under 5y, to varying levels of bias in input U5DR survey data. Each sensitivity value is a percentage of under 5y deaths that may have been missed during survey interviews.

Trends in the rate of people killed, by accessibility status of LGAs

Figure S15 displayed reported rates of people killed (combining the different insecurity monitoring project datasets with our reconstructed population denominators) by source and whether the LGA was fully accessible or not during any given month (accessibility was determined by us based on document review: see Table 1, main text).

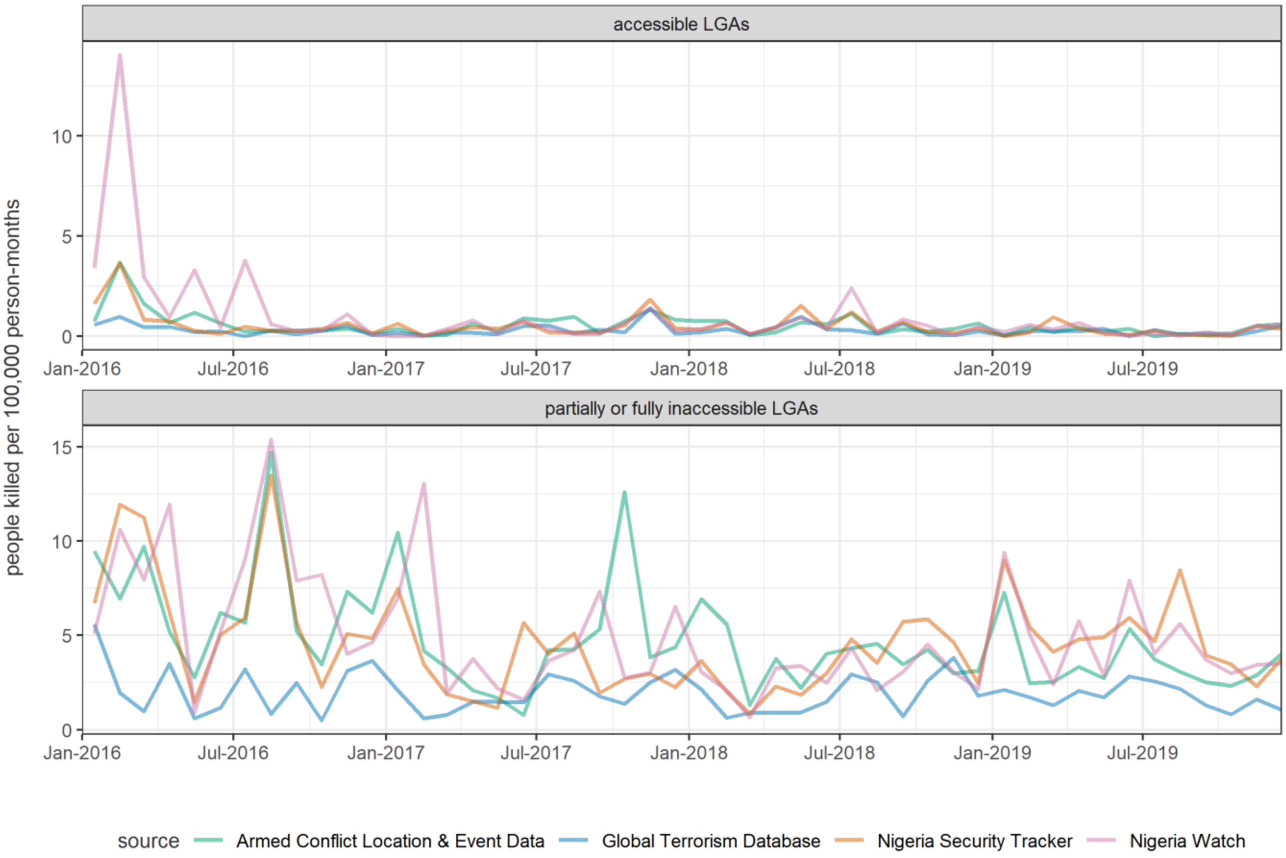

Figure S15. Trends in the monthly rate of people being killed according to each of the four insecurity monitoring projects for which data were available, and by whether LGAs were accessible or partially / fully inaccessible.

Sensitivity analysis: potential inaccessibility bias

Figure S16 shows how the total and excess estimated death toll varies if one assumes varying levels of bias in the estimated CDR for LGAs that were partly or completely inaccessible during any given month. As expected, results are highly sensitive to this potential bias in Borno state, where most LGAs were at least partially inaccessible during the analysis period.

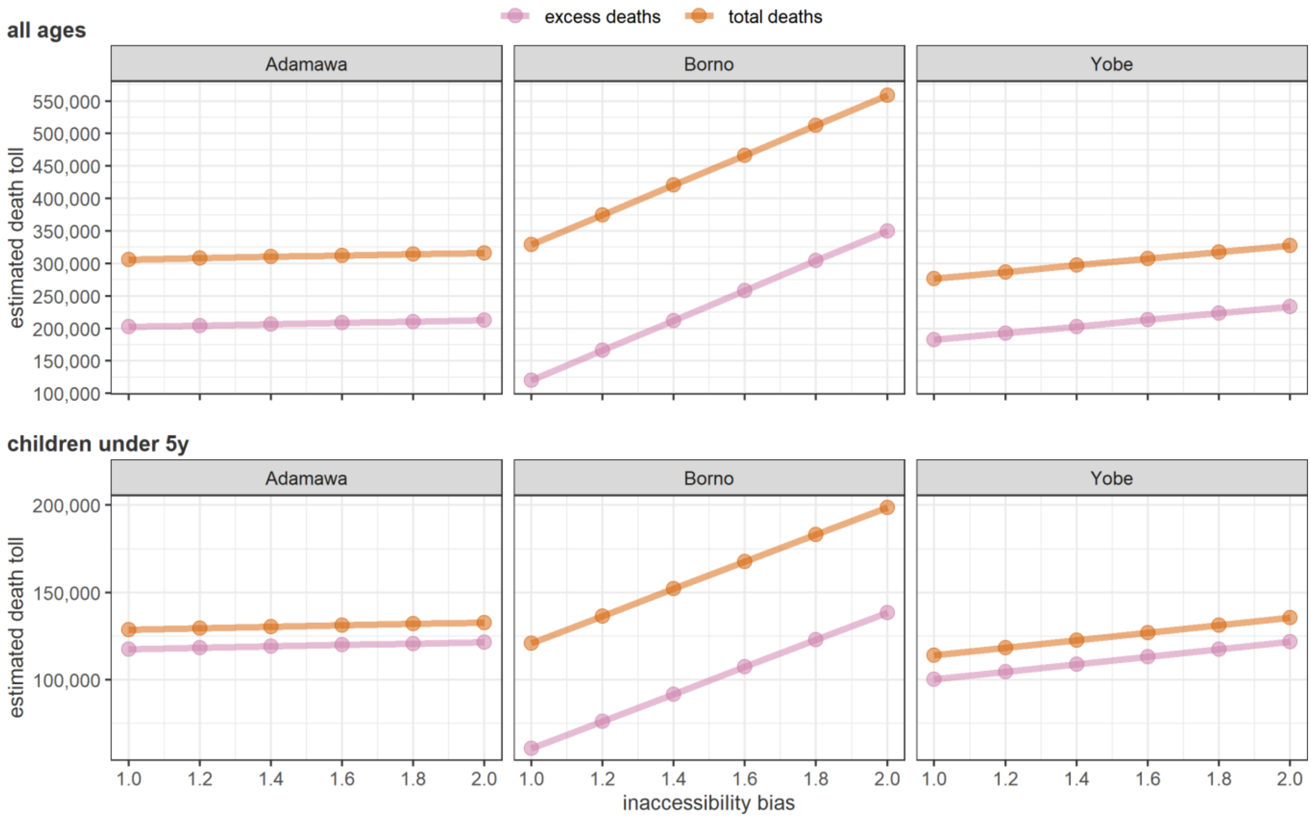

Figure S16. Estimated total and excess death toll, by age group and state, for increasing values of potential inaccessibility bias (each value is a multiplier for the estimated CDR, applied for LGA-months where accessibility was partial or none).

References

1. Nigeria - Internally displaced persons - IDPs - Humanitarian Data Exchange. https://data.humdata.org/dataset/idmc-idp-data-for-nigeria. Accessed 26 Apr 2023.

2. Erhardt J. Emergency Nutrition Assessment (ENA) Software for SMART. 2020.

3. Weber EM, Seaman VY, Stewart RN, Bird TJ, Tatem AJ, McKee JJ, et al. Census-independent population mapping in northern Nigeria. Remote Sensing of Environment. 2018;204:786–98.

4. Methodology: High Resolution Population Density Maps + Demographic Estimates. Facebook Data for Good. https://dataforgood.fb.com/docs/methodology-high-resolution-population-density-maps-demographic-estimates/. Accessed 23 May 2021.

5. Higgins J, Adamu U, Adewara K, Aladeshawe A, Aregay A, Barau I, et al. Finding inhabited settlements and tracking vaccination progress: the application of satellite imagery analysis to guide the immunization response to confirmation of previously-undetected, ongoing endemic wild poliovirus transmission in Borno State, Nigeria. Int J Health Geogr. 2019;18:11–11.

6. Abdelmagid N, Checchi F. Estimation of population denominators for the humanitarian health sector: Guidance for humanitarian coordination mechanisms. London: London School of Hygiene and Tropical Medicine; 2018.

7. Buuren S van, Groothuis-Oudshoorn K. mice: Multivariate Imputation by Chained Equations in R. Journal of Statistical Software. 2011;45:1–67.
